# Widespread Genetic Signals of Visual System Adaptation in Deepwater Cichlid Fishes

**DOI:** 10.1093/molbev/msaf147

**Published:** 2025-06-09

**Authors:** Julia I Camacho García, Milan Malinsky, Domino A Joyce, M Emília Santos, Grégoire Vernaz, Maxon J Ngochera, Hannes Svardal

**Affiliations:** Evolutionary Ecology Group, Department of Biology, University of Antwerp, Antwerp, Belgium; Institute of Ecology and Evolution, Department of Biology, University of Bern, Bern, Switzerland; Evolutionary and Ecological Genomics Group, University of Hull, Hull, UK; Department of Zoology, University of Cambridge, Cambridge, UK; Wellcome/Cancer Research UK Gurdon Institute, University of Cambridge, Cambridge, UK; Wellcome Sanger Institute, Wellcome Genome Campus, Hinxton, UK; Department of Fisheries Headquarters, P.O. Box 593, Lilongwe, Malawi; Evolutionary Ecology Group, Department of Biology, University of Antwerp, Antwerp, Belgium; Naturalis Biodiversity Center, Leiden, The Netherlands

**Keywords:** cichlid, visual system, ecological adaptation, evolutionary genomics

## Abstract

The light environment exerts a profound selection pressure on the visual system, driving morphological and molecular adaptations that may also contribute to species diversification. Here, we investigate the evolution and genetic basis of visual system diversification in deepwater cichlid fishes of the genus *Diplotaxodon*. We find that *Diplotaxodon* exhibit the greatest eye size variation among Lake Malawi cichlids and that this variation is largely uncoupled from phylogeny, with various nonsister species sharing similar eye sizes. Using a combination of genome-wide association analysis across nine *Diplotaxodon* species, haplotype-based selection scans, and transcriptome analysis, we uncover consistent and widespread signatures of evolution in visual pathways, centered on green-sensitive opsins and throughout the phototransduction cascade, suggesting coordinated evolution of eye size and visual molecular pathways. Our findings underscore the role of visual system diversification in niche specialization within deepwater habitats and offer new insights into visual system evolution within this extraordinary cichlid radiation.

## Introduction

Light is an important driver of evolutionary diversification of the visual sensory system, shaping species diversity through morphological and physiological changes (Cronin et al. 2014). In aquatic environments, where light attenuates exponentially with depth, this relationship can be specially pronounced, as organisms must compensate for extreme reductions in light intensity and shifts in spectral composition (Warrant and Locket 2004; Warrant and Johnsen 2013). One of the most conspicuous modifications to the visual system is an increase in eye size—a trait observed in many deepwater fishes and cephalopods, as well as in nocturnal vertebrates and insects (Warrant and Locket 2004; Land and Nilsson 2012). Larger eyes can capture more light by accommodating larger pupil apertures and lenses, thereby enhancing visual sensitivity in dimly lit environments (Land and Nilsson 2012).

Beyond eye size, visual adaptations occur across multiple levels of biological organization, including changes to lens properties, visual pigments (composed of a light-absorbing chromophore and an opsin protein; Bowmaker 2008), photoreceptor composition (rods and cones), and the phototransduction cascade (Carleton et al. 2020). These features often form suites of adaptations that broadly align with an organism's visual ecology. For example, in deepwater and nocturnal fishes, morphological features like enlarged eyes are often accompanied by other structural and molecular specializations, such as rod-only or rod-dominated retinas with opsins tuned to blue-green wavelengths, or elongated photoreceptors (Carleton et al. 2020; de Busserolles et al. 2020). Some of these mechanisms have been extensively studied in teleost fishes, providing valuable insights into how visual systems diversify in response to ecological pressures (Carleton et al. 2020; Musilova et al. 2021). Among these, cichlid fishes (family Cichlidae) have been particularly powerful as a model system due to their extraordinary visual diversity (Carleton 2009; Carleton et al. 2016; Carleton and Yourick 2020).

Cichlids, a species-rich group of freshwater fishes, strongly rely on vision for navigation, feeding, and mate choice—key factors in their rapid diversification (McGee et al. 2020; Santos et al. 2023). Differences in ecology and light environment have been linked to extensive visual pigment variation in cichlids (reviewed in Carleton 2009; Carleton and Yourick 2020), with depth-related visual adaptation playing crucial roles in their adaptive radiations in lakes Tanganyika (Ronco et al. 2021; Ricci et al. 2022, 2023), Victoria (Seehausen et al. 2008), Malawi (Hahn et al. 2017; Malinsky et al. 2018; Blumer et al. 2025), Masoko (Malinsky et al. 2015), and Barombi Mbo (Musilova, Indermaur et al. 2019) and contributing to ecological divergence and reproductive isolation among incipient species (Terai et al. 2006; Seehausen et al. 2008; Malinsky et al. 2015; Takuno et al. 2019). However, visual diversification beyond opsin tuning remains understudied, and the extent to which other components of cichlid visual systems have diversified remains largely unexplored. Here, we address this question by examining broad-scale visual adaptations in deepwater cichlids from Lake Malawi.

Lake Malawi, home to a cichlid radiation of over 800 extant species (Konings 2016), has deepwater habitats that extend from approximately 50 m to the anoxic layer at about 230 m (Eccles 1974). Of the seven lineages that make up this extraordinary radiation—usually referred to as “eco-morphological” clades (Malinsky et al. 2018)—two are adapted to the deepwater environment: a diverse group of ∼150 benthic species (the “deep benthic” clade), and the genus *Diplotaxodon* (Trewavas 1935), comprising ∼27 estimated species (nine described) of open-water planktivores and piscivores (Turner et al. 2004; Konings 2016; Stauffer et al. 2018). Previously, a radiation-wide scan for adaptive protein evolution (Malinsky et al. 2018), identified key visual genes (rhodopsin, green-sensitive opsins, and several other phototransduction genes) as outliers, with many of them exhibiting signals of parallel adaptation between the deep benthic clade and *Diplotaxodon*. Within *Diplotaxodon*, catch-depth records and marked variation in eye size suggest that diversifying selection may have contributed to differential visual adaptations. Notably, species restricted to greater depths (deeper than 50–80 m) tend to exhibit larger eyes than those occupying broader depth ranges that include shallower waters (20 to 30 m) (Turner et al. 2004). This raises the possibility of habitat partitioning and warrants the hypothesis that their visual systems have undergone adaptive evolution in response to ecological specialization. This idea is further supported by a restriction-site associated DNA sequencing (RADseq) study (Hahn et al. 2017) that identified candidate regions under divergent selection across four *Diplotaxodon* species, including genes related to visual perception (e.g. rhodopsin).

In this study, we investigate the evolution and genetic underpinnings of visual diversification in *Diplotaxodon* by integrating morphological and genomic data. We focus on eye size as a key trait of interest, given its high variability across cichlids and within this genus, and its general association with light availability and, more roughly, depth. While genetic variation linked to eye size may directly contribute to differences in this trait, it may also more broadly reflect adaptation to (visual) ecological niches beyond eye size itself. To contextualize eye size variation in *Diplotaxodon*, we examine morphological data from 1,334 individuals spanning 251 species across the Lake Malawi cichlid radiation. Performing genome-wide association (GWA) analysis for eye size, we identify many vision-related genes covering a broad functional spectrum. Enrichments for nonsynonymous substitutions, signals of natural selection, and cross-species differential expression of these genes support our hypothesis that visual adaptation contributed to diversification in *Diplotaxodon*. Furthermore, transcriptome analysis of eyes revealed expression differences in oxygen transport-related genes across species exhibiting differences in eye size, supporting depth-related adaptations beyond visual tuning. Overall, our study provides new insights into the genetic and phenotypic mechanisms underlying visual specializations in one of the most extreme environments into which cichlids have diversified.

## Results

### Large-Eye Size Variation Within and Across *Diplotaxodon* Species

**Fig. 1.**
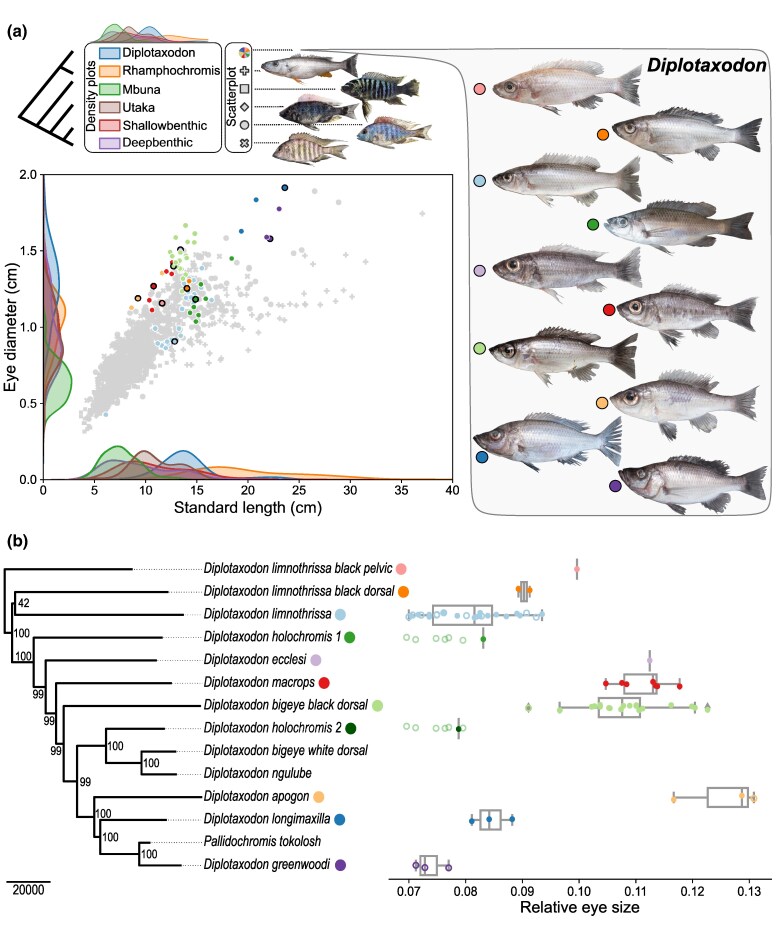
Eye size variation and genetic relationships in *Diplotaxodon*. a) Eye diameter versus standard length for 10 *Diplotaxodon* species (*N* = 75, colored circles) and members of the other five major eco-morphological clades of the Malawi cichlid radiation (+: Rhamphochromis, *N* = 62; □: Mbuna, *N* = 563; ○: Shallow benthic, *N* = 401; ×: Deep benthic, *N* = 170; ◊: Utaka, *N* = 63; grey markers). Kernel density estimate plots along the axes show the distributions of eye diameter and standard length per clade. Above, representative specimens from each clade (not to scale) and a diagram illustrating their phylogenetic relationships are displayed. The representative species are: *Rhamphochromis ferox* (Rhamphochromis), *Maylandia zebra* (Mbuna), *Otopharynx tetrastigma* (Shallow benthic), *Placidochromis hennydaviesae* (Deep benthic), *Copadichromis quadrimaculatus* (Utaka). For *Diplotaxodon*, one representative specimen per species is shown, with the corresponding data points outlined in black on the scatterplot. b) Neighbor joining (NJ) phylogeny based on whole-genome sequences of 13 *Diplotaxodon* species, including the monotypic genus *Pallidochromis* nested within the *Diplotaxodon* clade. Node labels show bootstrap support based on resampling distance matrices in 100 kb windows along the genome. On the right, relative eye sizes (eye diameter divided by standard length) of the individuals in (a); filled circles represent sequenced samples included in the genome-wide association analysis for eye size (*N* = 53), while empty circles represent phenotyped specimens without sequences, assigned to a species based on morphological inspection. The boxes indicate the three quartile values of the distribution, and the whiskers are the minimum and maximum values. Values outside 1.5× the interquartile range are represented as diamonds. Given that the two sequenced *D*. “holochromis” individuals are not monophyletic in the NJ tree, it is not possible to confidently assign nonsequenced samples morphologically identified as *D.* “holochromis” to either group; however, to show eye size variation, these samples are included along both species (“holochromis 1/2”) (duplicated data points = green empty circles with increased transparency).

To assess eye size variation within the Lake Malawi cichlid adaptive radiation, we measured eye diameter across 1,334 individuals of six eco-morphological clades (supplementary Data S1 and S2, Supplementary Material online). As expected, eye size scaled positively with body standard length (Fig. 1a), although this relationship was not equally strong across eco-morphological groups (supplementary fig. S1a, Supplementary Material online). Therefore, we used linear regression to adjust for body size within each clade, revealing that the deepwater pelagic clade of *Diplotaxodon* stood out as having the largest within-group variation in eye size (supplementary fig. S1b and c, Supplementary Material online; mean residual *Diplotaxodon* = 0.17, other groups = from 0.05 to 0.1).

### Parallel Evolution of Eye Size in *Diplotaxodon*

Given uncertainties regarding species assignments and relationships within *Diplotoxodon* (Turner et al. 2004; Malinsky et al. 2018), we used whole-genome data of 79 *Diplotaxodon* individuals from 13 species to provide evolutionary context for investigating differences in eye size. Constructing a neighbor joining (NJ) tree based on pairwise genetic distances (Fig. 1b; supplementary fig. S2, Supplementary Material online), we found that all individuals assigned to the same species based on morphological examination formed monophyletic groups, except for our two sequenced *D.* “holochromis”, which clustered separately from each other and will be treated as distinct taxonomic entities (*D.* “holochromis 1” and *D.* “holochromis 2”) in the following.

Across all *Diplotaxodon* individuals, eye diameter varied between 7% and 13% of standard length (Fig. 1b). Interestingly, we found that this variation in relative eye size was largely decoupled from the phylogeny. Specifically, Pagel's lambda (λ), a measure of phylogenetic signal with respect to a Brownian model of trait evolution (Pagel 1999), was close to zero (Pagel's λ = 6.6 × 10^−5^), indicating a large degree of independence of eye size from the species evolutionary relationships. Given the low sample size for some species and a lack of a fully resolved phylogeny, we interpret this value with caution. However, it is clear that various nonsister lineages share similar eye sizes across the phylogeny, for example, *D. macrops*, *D.* “bigeye black dorsal”, and *D. apogon* have a “large-eye” phenotype and *D. limnothrissa*, and *D.* “holochromis 1/2” have a “small-eye” phenotype (Fig. 1b; note that despite their small relative eye size, we will generally not consider the predatory *D. longimaxilla* and *D. greenwoodi* in the latter category as their much larger size and distinctive general body shape set them apart from the rest). Moreover, the correlation between genetic distance and eye size differences is relatively low (*R*^2^ = 0.15, *P* = 4.7 × 10^−42^), much lower than, for example, the correlation between genetic distance and body shape differences (*R*^2^ = 0.55, *P* = 5.8 × 10^−201^) (supplementary fig. S3, Supplementary Material online). Overall, the decoupling of this trait from phylogeny and genetic distance suggests that eye size has evolved in parallel in distinct lineages and creates an opportunity for investigating which genetic variants are linked to variation in the trait.

### Genome-Wide Significant Eye Size Associations

**Fig. 2.**
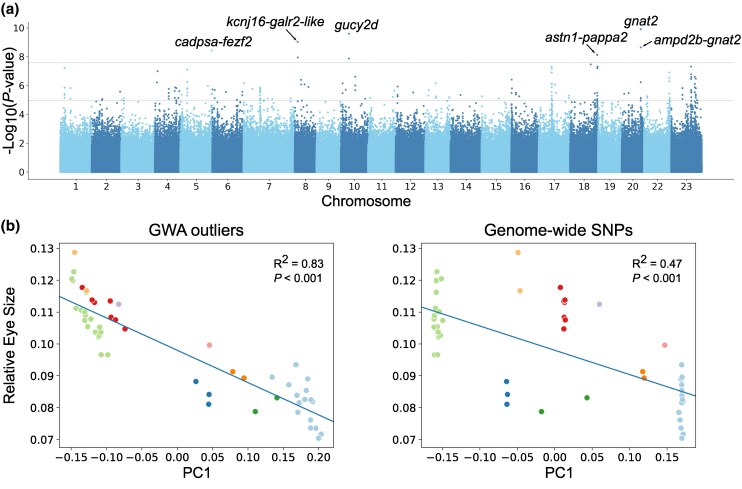
Genome-wide association analysis for eye size in *Diplotaxodon*. a) Manhattan plot showing genome-wide associations for relative eye size across nine *Diplotaxodon* species (*N* = 53). Top horizontal line: Bonferroni-corrected threshold at *FWER* < 0.05; bottom horizontal line (dashed): cutoff 0.01% most significant associations. Genes annotated to Bonferroni-significant SNPs are shown as a single name (if exonic) or two names separated by a dash (intergenic); b) Principal component (PC) 1 against relative eye size for PC analyses summarizing genetic variation on GWA outliers (190 SNPs; left) and genome-wide (MAF > 5%, 377,273 SNPs; right). In both plots, a regression line and R-squared of relative eye size ∼ PC1 are shown. Species color keys as in Fig. 1.

To investigate genetic variation associated with the observed differences in eye size, we performed a GWA analysis for relative eye size, focusing on the *Diplotaxodon* individuals with both whole-genome sequences and phenotypic data (filled circles in Fig. 1b, *N* = 53). Despite the moderate sample size, we identified 10 single nucleotide polymorphisms (SNPs) associated with eye size with genome-wide significance (Bonferroni FWER < 0.05) on five chromosomes (Fig. 2a).

The two strongest associations, on chromosomes 10 and 20, corresponded to nonsynonymous mutations in the genes *gucy2d* (*P* = 2.57 × 10^−10^) and *gnat2* (*P* = 1.26 × 10^−10^) (Fig. 2a), both coding for proteins of the cone photoreceptors in the retina, where they play essential roles in phototransduction—the process by which photons of light are converted into electrical signals (Lamb and Curtin 2022). Among the remaining eight Bonferroni-significant SNPs, there was an additional nonsynonymous mutation mapping to *gucy2d*. The rest were located in intergenic regions nearby the genes *kcnj16*, LOC113027815 (*galr2*-like), *ampd2b*, *gnat2*, *cadpsa*, *fezf2*, *pappa2,* and *astn1*. These genes participate in a range of functions, including neurotransmission and neurogenesis (*galr2*, *fezf2*, *cadpsa*, *astn1*), energy metabolism (*ampd2b*), and homeostasis (*kcnj16*) (Stelzer et al. 2016; The UniProt Consortium 2023). Beyond the conservative Bonferroni-threshold, there were several other association peaks that might correspond to biologically relevant signals (Fig. 2a). Therefore, to include these in further analyses we considered as outlier SNPs the 0.01% most significant associations, corresponding to 190 SNPs with an eye size association of *P* < 0.00001 (“GWA outliers”).

The linear mixed model used to estimate GWA accounts for population stratification (Price et al. 2010; Zhou and Stephens 2012). To verify that the GWA signals were not driven by residual stratification in our highly structured sample set, we used principal component (PC) analysis to compare genome-wide sample genetic variation to that among GWA outlier SNPs. We found that GWA outliers separated samples along PC1 by eye size to a larger degree than would be expected by phylogeny alone (Fig. 2b). Specifically, *D.* “holochromis 1” and *D.* “holochromis 2” were closer to samples of *D. limnothrissa* spp. (collectively referred to as *D. limnothrissa* complex) than to their sister species, which formed a separate cluster. A linear regression confirmed that PC1 from GWA outlier SNPs explain variation in eye size to a much greater extent (R^2^ = 0.83, *P* < 0.0001) than PC1 from genome-wide variation (R^2^ = 0.47, *P* < 0.0001) (Fig. 2b).

### Eye Size-Associated Loci Harbor Vision-Related Genes

To gain insight into the biological functions of the genetic variants associated with eye size, we examined whether genes mapping to 50 kb regions centered on each of the GWA outliers were enriched for specific gene ontology (GO) terms. We found 104 genes annotated to these regions, and significant enrichment of 30 GO terms in the “biological process” category (Fisher's exact test, *P* < 0.05; supplementary table S3, Supplementary Material online), with the terms “cone photoresponse recovery” (GO:0036368, *P* = 0.0006), “detection of visible light” (GO:0009584, *P* = 0.003) and “visual perception” (GO:0007601, *P* = 0.004) as the most highly significant. Among the genes in these categories was *gnat2*, which corresponds to the strongest GWA (Fig. 2a), together with *arr3a*, *cnga3a*, *grk7a,* and *rcvrn3* (supplementary table S3, Supplementary Material online). Like *gnat2*, these genes are integral to the phototransduction cascade, contributing to the activation, termination, and modulation of the visual response (Pugh and Lamb 2000; Lamb and Curtin 2022).

Given the high representation of visual transduction genes within GWA outlier loci, we followed up on this result with a manual examination of the functional annotation of GWA hits (see Methods), revealing three additional phototransduction genes: *gucy2d* (reported above as Bonferroni-significant, Fig. 2a), *gngt2*, and *pde6h*-like. Lowering the GWA significance cutoff from 0.01% to 0.025% (475 SNPs, *P* < 5 × 10^−5^) further revealed the presence of the cone arrestin *arr3b*, rhodopsin (*rh1*), and the green-sensitive opsin *rh2aβ* within GWA loci. However, as these genes do not meet the established 0.01% cutoff, they are noted here but not considered in downstream analyses. Besides phototransduction, we found other examples of genes implicated in visual functions annotated to GWA loci, namely eye development (*pawr*; Acharya et al. 2011), retina regeneration (*ascl1a*; Džulová et al. 2022), and photoreceptor synaptic structure (*pappaa*; Miller et al. 2018) (see supplementary Data S5, Supplementary Material online for the full annotation of GWA outlier SNPs).

### Excess of Nonsynonymous Variants Among GWA Outliers

Focusing on the 190 (0.01% most significant) GWA outlier SNPs associated with eye size, we observed that 34 of these were amino acid changing, which was 9.6 times higher than expected by chance (empirical permutation *P* < 0.001; supplementary fig. S4, Supplementary Material online). Of these nonsynonymous SNPs, 12 mapped to 5 of the phototransduction genes reported above, while the remaining 22 were located in other genes, some of which are potentially relevant to depth adaptation and muscle function (Table 1). The presence of genes with nonvisual functions among the candidates may reflect broader species-level divergence related to their habitat depths and/or ecological niches. This suggests that we might have identified causal SNPs with an adaptive function.

**Table 1:** Genes annotated to GWA outliers putatively relevant for deepwater adaptation and skeletal muscle function

Gene symbol	Description	PT	Role(s)
**gnat2**	G protein subunit alpha transducin 2	Yes	Involved in the activation of phototransduction in cone photoreceptors. Stimulates the coupling of the opsin and cGMP-phosphodiesterase during visual impulses^a^. Human orthologs associated with achromatopsia and cone dystrophy^b^.
gngt2	G protein subunit gamma transducin 2	Yes	Involved in the activation of phototransduction in cone photoreceptors. Required for GTPase activity, replacement of GDP by GTP, and G protein-effector interaction^c^.
**LOC113021214** (pde6h-like)	Retinal cone rhodopsin-sensitive cGMP 3′,5'-cyclic phosphodiesterase subunit gamma-like	Yes	Involved in the activation of phototransduction in cone photoreceptors. Mediates the transmission and amplification of the visual signal by catalyzing the conversion of cGMP to GMP, causing the nucleotide-gated channels to close^a^. Human orthologs associated with retinal cone dystrophy and achromatopsia^b^.
**cnga3a**	Cyclic nucleotide-gated channel subunit alpha 3a	Yes	Involved in the activation of phototransduction in cone photoreceptors. Regulates the flow of cations into the cone, modifying its electrical charge and generating an electrical signal (interpreted as vision in the brain)^a^.
**gucy2d**	Retinal guanylyl cyclase 2-like	Yes	Regulates phototransduction by mediating the synthesis of cGMP from GTP in rods and cones^a^. Human orthologs associated with Leber congenital amaurosis and cone-rod dystrophy^b^.
grk7a	G protein-coupled receptor kinase 7a	Yes	Involved in the termination of phototransduction in cone photoreceptors by deactivating cone opsins via phosphorylation^a^.
**arr3a**	Arrestin 3a, retinal (X-arrestin)	Yes	Involved in the termination of phototransduction in cone photoreceptors by binding to the phosphorylated opsin^a^.
rcvrn3	Recoverin-like	Yes	Modulates light sensitivity of the photoreceptors in dark and dim conditions. In low light levels, inhibits GRK activity, prolonging opsin activation^a^. Facilitates the detection of change and motion in bright light^b^.
**si:ch1073-13h15.3**	Putative all-trans-retinol 13,14-reductase (retsat)	No	May play a role in the metabolism of vitamin A (the visual pigment chromophore) in zebrafish^b^. Involved in apocarotenoid metabolism in birds, mediating SWS2 spectral tuning^d^. Changes in vitamin A signaling during development mediated by *retsat* could be implicated in changes in eye size and abnormal eye morphology in Atlantic haddock^e^. Implicated in hypoxia adaptation in Qinghai-Tibet Plateau mammals^f^.
**trhde.2**	Thyrotropin releasing hormone degrading enzyme tandem duplicate 2	No	Part of the thyroid hormone (TH) pathway, where it cleaves and inactivates the neuropeptide thyrotropin-releasing hormone. TH plays a role in photoreceptor development^g^ and visual plasticity by modulating opsin expression and chromophore exchange^h,i^. The gene trhde is involved in high-altitude adaptation in Ethiopian sheep^j^.
**LOC113022044** (*igfn1*-like)	Immunoglobulin-like and fibronectin type III domain containing protein 1	No	Predicted to be involved in homophilic cell adhesion, retina layer formation and synapse assembly^b^. Human ortholog associated with polypoidal choroidal vasculopathy, a disease affecting the layer of blood vessels in the choroid^k^.
**cahz**	Carbonic anhydrase-like	No	Involved in carbon dioxide transport and hypotonic salinity response. Contributes to acid–base homeostasis, fluid balance, and the formation of aqueous humor in the eye, influencing its water content. May enhance tissue oxygen delivery in teleosts^l^. Suggested to play a role in oxygenation of the inner retina, potentially contributing to the adaptive evolution of vision in teleost fish^m^.
pappaa	Pappalysin 1 Pregnancy-associated plasma protein aa	No	Encodes a metalloendopeptidase known to activate insulin-like growth factor 1 (IGF1)^b^. Regulates photoreceptor synaptic structure and function of cone synapses to transmit light-offset information. Pappaa mutant zebrafish have impaired responses to light offset^n^.
**ttn.2**	Titin, tandem duplicate 2	No	Structural component of muscle involved in heart contraction, myofibril assembly and striated muscle tissue development^c^.
**neb**	Nebulin	No	Involved in muscle fiber development. May play a role in maintaining the structural integrity of sarcomeres and the membrane system associated with the myofibrils^c^.
pvalb*	Parvalbumin	No	Parvalbumins are calcium ion-binding proteins involved in muscle relaxation after contraction. Expressed in other tissues, where its function is not well understood^o,p^.

It is noted if the genes are involved in the phototransduction molecular pathway (“PT”) or not. Genes with nonsynonymous GWA outliers are highlighted in bold.

*pvalb is the generic name used to refer to genes encoding parvalbumin, of which multiple copies are present in teleosts (Mukherjee et al. 2021). Several copies are present in the annotation of GWA outliers, here collectively referred to as *pvalb* for simplicity.

References: ^a^Pugh and Lamb (2000); ^b^Stelzer et al. (2016); ^c^The UniProt Consortium (2023); ^d^Toomey et al. (2016); ^e^Lie et al. (2019); ^f^Xu et al. (2021); ^g^Ng et al. (2001); ^h^Karagic et al. (2018); ^i^Volkov et al. (2020); ^j^Edea et al. (2019); ^k^Wen et al. (2018); ^l^Rummer and Brauner (2011); ^m^Damsgaard et al. (2020); ^n^Miller et al. (2018); ^o^Mukherjee et al. (2021); ^p^Permyakov and Uversky (2022).

Following the discovery of an excess of nonsynonymous variation, we next investigated whether evolutionary change happened in the big-eyed or small-eyed species by inspecting the derived allele frequencies (AF) at these nonsynonymous sites. Overall, across the 34 nonsynonymous GWA outliers, there was no consistent pattern of whether the derived allele was present at high frequency in big or small-eyed species (chi-square *P* = 0.8). However, when considering only phototransduction genes, we observed a striking difference: in 11 out of the 12 nonsynonymous GWA outliers mapping to phototransduction genes the derived allele was fixed or nearly fixed in the big-eyed species, but absent or at low frequency in small-eyed species (chi-square *P* < 0.001; supplementary table S4, Supplementary Material online). The opposite pattern was found for genes with putative skeletal muscle functions for which seven out of eight genes showed high-frequency derived alleles in small-eyed species (chi-square *P* = 0.01).

Overall, our findings confirm that eye size-linked genetic variants are enriched for functional alleles and that qualitatively different molecular functions have been under adaptive evolution in big-eyed compared to small-eyed *Diplotaxodon*.

### Recent Divergent Selection Around Candidate Genes for Depth Adaptation

**Fig. 3.**
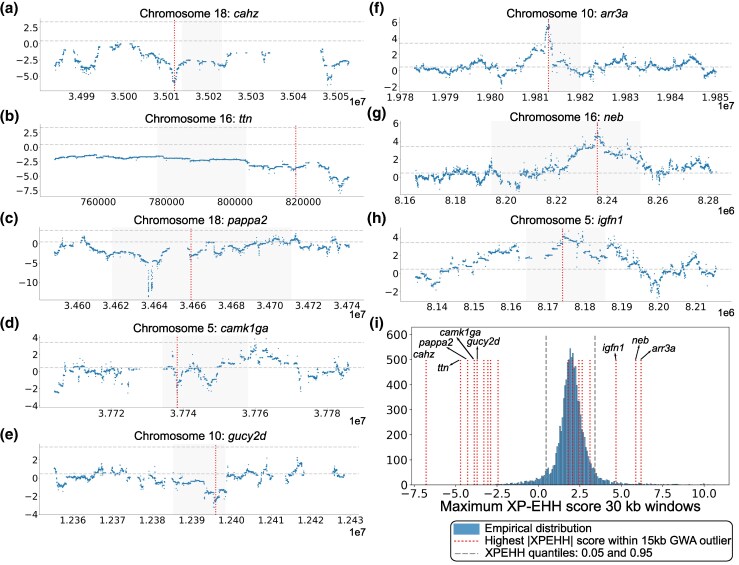
Evidence of divergent selection in candidate genes between *D. limnothrissa* (*N* = 33) and *D.* “bigeye black dorsal” (*N* = 20). Extreme negative (empirical genome-wide quantile < 0.05; panels a–e) and positive (quantile > 0.95; panels f–h) XP-EHH scores (y-axis) within 15 kb from nonsynonymous GWA outliers are marked with red vertical lines. For negative outliers, only the top five genes are shown. Horizontal dashed lines indicate the 0.05 and 0.95 quantiles (see panel i). Grey shading highlights the coding region of each gene; i) Empirical distribution of maximum XP-EHH scores in random 30 kb windows across the genome from 10,000 iterations, with red vertical lines marking the highest XP-EHH score within 15 kb from nonsynonymous GWA outliers for eye size.

To further test for recent divergent selection between small and large-eyed *Diplotaxodon*, we applied the cross-population extended haplotype homozygosity (XP-EHH) statistic (Sabeti et al. 2007) to the two representative species with the largest sample sizes—the small-eyed species *D. limnothrissa* (*N* = 33) and the large-eyed species *D. “*bigeye black dorsal” (*N* = 20). We focused specifically on genes harboring nonsynonymous GWA outliers, which we defined as candidate genes. We considered XP-EHH scores within 15 kb from nonsynonymous GWA outliers as evidence of selection if they ranked in the top 5% (quantile > 0.95) or bottom 5% (quantile < 0.05) of the genome-wide distribution of XP-EHH scores in 30 kb windows. Based on this definition, we found signals of selection around 11 of the 17 examined genes (Fig. 3).

Negative XP-EHH scores are suggestive of positive selection in the big-eyed species (Fig. 3a–e) while positive scores indicate selection in the small-eyed species (Fig. 3f–h). The strongest signal for *D*. “bigeye black dorsal” (XP-EHH score −6.8; smaller than the most negative score in any of the 10,000 genome-wide control windows; Fig. 3i) was detected downstream of the gene *cahz* (Fig. 3a and i), which encodes a carbonic anhydrase, an enzyme with a role in tissue oxygen delivery in teleosts (Rummer and Brauner 2011) (Table 1). For *D. limnothrissa*, the strongest selection signal (score 6.2, 0.99 quantile) was found immediately upstream of the gene *arr3a* (Fig. 3f and i). This gene is expressed in cone photoreceptor cells and codes for arrestin 3a, which mediates the termination of phototransduction (Pugh and Lamb 2000) (Table 1). While our initial outlier detection analysis was restricted to windows containing nonsynonymous GWA outliers, in some instances XP-EHH peaks were present in the same genes but outside of these windows. For example, we found two peaks of extreme negative scores (XP-EHH < −12; more negative than any control window; Fig. 3c), suggesting positive selection in the big-eyed species, within the coding region of *pappa2* situated 22 kb away from the nonsynonymous GWA outlier. Another particularly interesting pattern was present around the gene *ttn*, for which negative XP-EHH scores span sites across > 50 kb suggesting that selection acted rapidly or on multiple targets, or that the region lacks recombination (Fig. 3b).

### Selective Sweep in the Promoter Region of *arr3a*

**Fig. 4.**
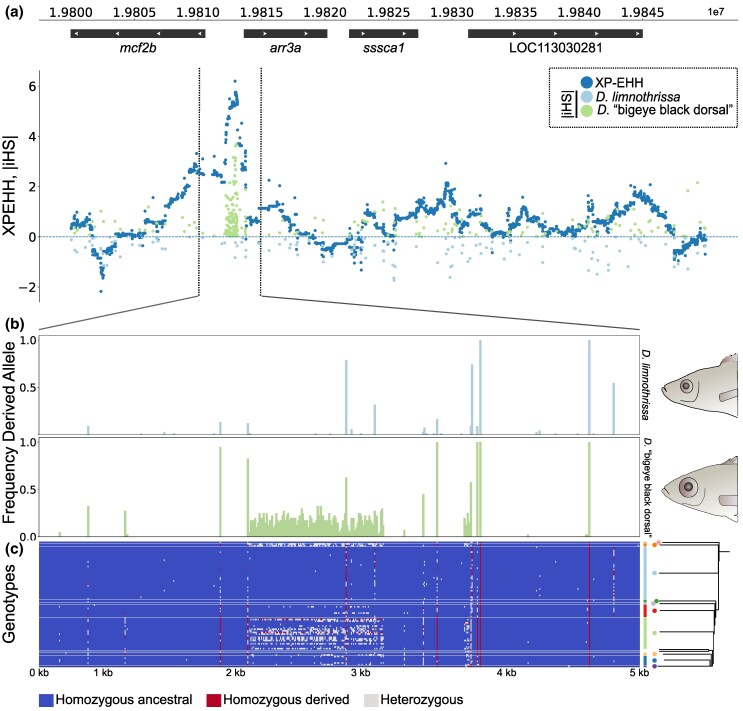
Evidence of selective sweep in the putative promoter region of the vision gene *arr3a*. a) Overlap of cross-population extended haplotype homozygosity (XP-EHH, dark blue dots) and absolute integrated haplotype scores (iHS) in *D.* “bigeye black dorsal” (green, positive axis) and in *D. limnothrissa* (light blue, negative axis) in a 50 kb region containing the *arr3a* locus. b–c) Allele frequencies (b) in *D. limnothrissa* (*N* = 33) and *D.* “bigeye black dorsal” (*N* = 20) and genotypes (c) in 10 *Diplotaxodon* species across a 5 kb region spanning the XP-EHH and iHS signal. Genotypes are sorted by phylogeny.

To complement the cross-population selection scan (XP-EHH), we used an additional statistic, the integrated haplotype score (iHS), which provides evidence of recent or ongoing selective sweeps within species (Voight et al. 2006). This analysis revealed a strong signal of selection in *D.* “bigeye black dorsal” immediately upstream of the transcription start site (TSS) of the vision-related *arr3a*, almost perfectly aligned with the XP-EHH peak around this gene (Fig. 4a). The overlap between the two statistics is seemingly contradictory, as they indicate selection acting in big-eyed species (iHS) and small-eyed species (XP-EHH). Such a pattern could be explained if this region had experienced historical selection in both populations, differing in nature and timing. For example, high XP-EHH scores could be reflecting an older selection event in *D. limnothrissa*, while the iHS signal could be pointing at a recent or partial selective sweep in *D.* “bigeye black dorsal”, with different alleles being favored in each species as expected under disruptive ecological selection (Maynard 1962). Consistent with this, we found a haplotype of approximately one kilobase containing derived alleles at intermediate frequency in *D.* “bigeye black dorsal”, with some of the alleles also present in other species (Fig. 4b and c). Most derived alleles in this region occur exclusively in heterozygous state (Fig. 4c). To confirm that this pattern was not caused by misalignment of an unresolved duplication, we checked coverage and mapping quality (MQ), concluding that no duplication was present (supplementary fig. S5, Supplementary Material online). We therefore suggest that this pattern is either the result of a recent, ongoing selective sweep or caused by a combination of balancing selection and suppressed recombination. In either case, our findings point to complex, ongoing selection on the promoter region of *arr3a*, likely affecting the expression of this key gene within the phototransduction pathway.

### Differential Gene Expression Between Big and Small-Eyed *Diplotaxodon*

While nonsynonymous variants were overrepresented among GWA outliers, the vast majority (169 of 190) were noncoding, potentially reflecting gene regulatory changes contributing to trait evolution (Hoekstra and Coyne 2007; Wray 2007). Therefore, to complement the GWA analyses, we examined gene expression differences across whole-eye transcriptomes between small-eyed (*D. limnothrissa* complex; *N* = 5) and big-eyed (*D.* “macrops black dorsal” + *D.* “macrops offshore”; *N* = 5) species. With this approach, we targeted not only genes expressed in the retina, such as phototransduction genes, but also genes expressed in other ocular tissues (e.g. lens, choroid, vitreous, blood vessels), capturing additional functions that could be relevant for depth adaptation.

**Fig. 5.**
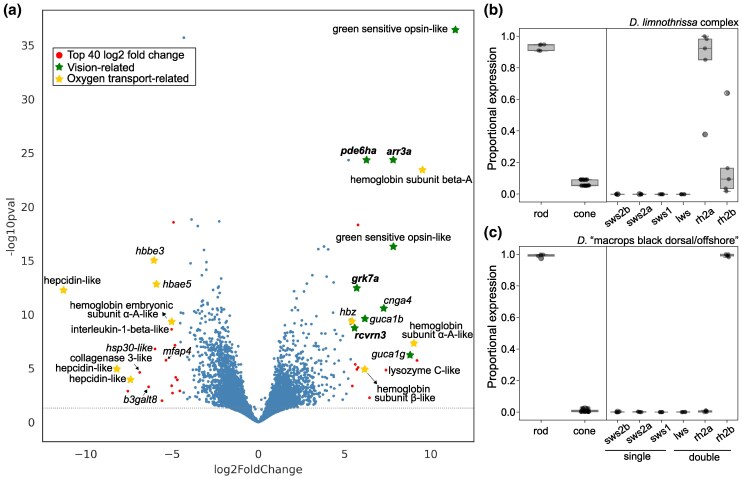
Differential gene expression analysis between small and large-eyed *Diplotaxodon* and opsin expression profiles. a) Gene expression differences (log2 fold change) across whole-eye transcriptomes of small-eyed species of the *D.* “limnothrissa complex” (*N* = 5) and big-eyed species of *D.* “macrops black dorsal/offshore” (*N* = 5) plotted against the -log10 *P*-value (Wald test, adjusted *P*-value after Benjamini–Hochberg correction). Each dot represents a gene, and the horizontal line indicates the *P* = 0.05 cutoff. The top 40 most differentially expressed genes (adjusted *P* < 0.05, absolute log2FC > 4.5) are marked with red dots and colored stars. Vision-related genes (based on the *A. calliptera* annotation, fAstCal1.2), are highlighted with green stars, and oxygen transport genes with yellow stars. Candidate genes that overlap with the genomic analysis are indicated in bold; b, c) Opsin gene expression in both groups, shown as rod opsin (*rh1*) and cone opsin expression relative to total opsin expression (left panel), and individual cone opsin expression relative to total cone opsin expression (single: *sws1*, *sws2a*, *sws2b*, and double cones: *rh2a*, *rh2b*, *lws*). Expression of the two *rh2a* copies is combined into a single value. Boxplots show the first and third quartiles (boxes), the median (central line), and the minimum and maximum values (whiskers). Outliers (values outside 1.5× the interquartile range) are shown as diamonds.

Out of 20,299 analyzed genes, 4,648 were differentially expressed (DE) (adjusted *P* < 0.05) between the two groups. Among the 40 most highly DE genes (absolute log2FC > 4.5, corresponding to a fold change in expression > 23; supplementary table S5, Supplementary Material online) there were two green-sensitive opsins and six additional genes of the phototransduction pathway (Fig. 5a). Four of these eight genes overlapped with the GWA candidates and, interestingly, all of them were overexpressed in the small-eyed species relative to the big-eyed species. Of these, *arr3a*, the promoter region of which we have shown to be under putative recent selection (Fig. 4), ranks sixth in the list of genes overexpressed in *D. limnothrissa* (supplementary table S5, Supplementary Material online). This further supports the idea that expression changes in *arr3a* play a role in ecological specialization between small and big-eyed *Diplotaxodon*. Besides vision-related genes, we found seven highly DE hemoglobin subunit genes (Fig. 5a; supplementary table S5, Supplementary Material online) and three hepcidin genes that were underexpressed in the *D. limnothrissa* group relative to the big-eyed group (Fig. 5a). Hepcidin mediates the uptake and release of iron—an essential component of hemoglobin and myoglobin—and has been shown to be downregulated in response to hypoxia (Nemeth and Ganz 2006).

### Expression Profile of Visual Opsin Genes

The subset of visual opsin genes expressed by a species—referred to as their “visual palette”—is crucial for determining spectral sensitivity and often correlates with ecological factors such as the light environment and foraging and mating behaviors (Carleton et al. 2016). To characterize the visual opsin repertoire used by small and big-eyed *Diplotaxodon*, we examined opsin gene expression values normalized as transcript per million (TPM) from the eye transcriptomes.

Rhodopsin (*rh1*) expression accounted for the majority of opsin gene expression in both groups (Fig. 5b and c). A high rod:cone opsin gene expression ratio is expected due to the much higher density of rods relative to cones in cichlid retinas (Braekevelt et al. 1998), but the extreme prevalence of rhodopsin expression observed (median [range]: 94.5% [90.8% to 94.8%] and 99.3% [97.5% to 99.9%] of total opsin expression in *D. limnothrissa* and *D.* “macrops black dorsal/offshore”, respectively) is likely reflecting evolutionary adaptation to low light in the deepwater environment shared by both species groups (Carleton et al. 2020). Consistent with this, cone opsin expression was dominated by green-sensitive opsins (*rh2*), while expression of long (*lws*) and short-wavelength sensitive (*sws1*, *sws2a*, *sws2b*) opsins was virtually absent in both species (TPM < 1, accounting for < 0.1% of total cone opsin expression; Fig. 5b and c; supplementary fig. S6, Supplementary Material online). While the small-eyed species expressed both *rh2a* and *rh2b* (90.6% [37.2% to 98.2%] and 9.4% [1.8% to 62.8%] of total cone opsin expression, respectively), the repertoire appeared more restricted in the big-eyed species, with only *rh2b* accounting for 99% [98.2% to 100%] of cone opsin expression (Fig. 5c). These differences in relative green opsin expression between small and big-eyed *Diplotaxodon* suggest fine-tuning of vision to their respective depth ranges within otherwise similar “deepwater visual palettes”.

## Discussion

Vision is essential for feeding, recognizing conspecifics, avoiding predators, and identifying potential mates in many organisms (Land and Nilsson 2012; Cronin et al. 2014). As a consequence, depth-related gradients of light attenuation are commonly associated with niche diversification involving both morphological and molecular adaptations of the visual system (Land and Nilsson 2012). In this study, we examined eye size variation and associated molecular changes to explore some of the adaptations that allowed the genus *Diplotaxodon* of the Malawi cichlid adaptive radiation to diversify in deepwater habitats.

Beyond the general trend for large eyes in *Diplotaxodon—*a phenotype common in deepwater fishes (Warrant and Locket 2004)—we found that *Diplotaxodon* also exhibit significant variability in relative eye size, exceeding that of any other eco-morphological clade within the radiation. This variation occurs both within and between species. Intraspecific variation may reflect phenotypic plasticity or differences between sexes and ontogeny (Meuthen et al. 2018; Svanbäck and Johansson 2019). However, much of this variability is interspecific, consistent with taxonomic reviews of the genus highlighting eye size as a distinguishing feature between some species (Turner et al. 2004).

Eye size is a complex trait shaped by multiple ecological and evolutionary forces, including diet, habitat complexity, developmental constraints and phylogenetic history (Niven and Laughlin 2008; de Busserolles et al. 2013; Lisney et al. 2020). *Diplotaxodon* are open-water zooplanktivores and predators of small fish, differing little in diet or general habitat preference aside from their presumed depth distributions (Turner et al. 2004). Although the challenges in accessing deepwater habitats limit detailed knowledge of their specific ecologies, trawl surveys and artisanal fishery data suggest that *Diplotaxodon* species with smaller eyes occupy broader depth ranges (∼20 to 220 m) compared to a restriction to greater depth (∼50 to 200 m) in species with larger eyes (Turner et al. 2004; Genner and Turner 2012), except for possible nocturnal migrations to the surface (Thompson et al. 1996). The steep decline in light intensity with depth likely results in a sharp contrast in the light environments experienced by species across even relatively small depth differences, suggesting that interspecific differences in eye size may, at least in part, reflect distinct visual demands associated with depth. The repeated evolution of eye size in several nonsister lineages, as revealed by our inferred genetic relationships, further supports this hypothesis. We therefore investigated eye size variation as a potential proxy for molecular (visual) adaptations in this group.

The lack of strong phylogenetic signal in eye size variation is important because it increases the power to distinguish functional genetic variation responsible for shared molecular adaptation from linked variation and neutral species divergence. And indeed, despite a relatively small sample size, we found genome-wide significant associations with eye size on five chromosomes, with a range of additional evidence (e.g. overrepresentation of amino acid changing mutations) suggesting that we were able to identify functional genetic variation.

Although none of the identified variants are known to directly control eye size, some could still play an indirect role in eye development. Notably, one of the 10 Bonferroni-significant SNPs fell in an intergenic region adjacent to the gene *fezf2*. This gene encodes a transcription factor part of a regulatory network involved in early brain patterning, during which the regions that give rise to the forebrain and the eye field compete for developmental territory (Sylvester et al. 2010; Patil et al. 2021). Divergence in this network has been shown to underlie differences in forebrain structure between rocky and sand-dwelling cichlids, with potential implications for behavior and sensory processing (Sylvester et al. 2010; Patil et al. 2021). These findings raise the possibility of an effect of *fezf2* in eye size, potentially through its role in early neural patterning, though establishing a causal relationship would require experimental validation.

**Fig. 6.**
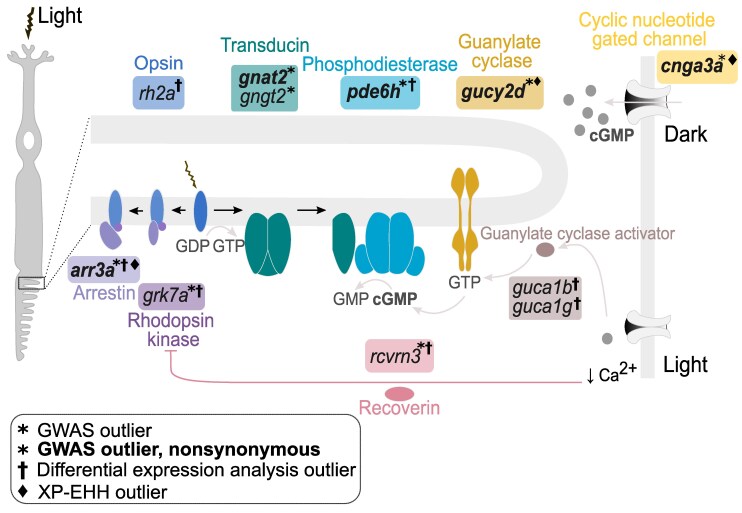
Schematic of the vertebrate phototransduction cascade. The main components and events are summarized. Briefly, light activates the opsin protein, which stimulates the alpha subunit of transducin, leading to the activation of phosphodiesterase. This enzyme hydrolyzes cyclic guanosine monophosphate (cGMP), lowering its concentration in the cell and causing the cyclic gated nucleotide channels to close. This results in a reduced influx of cations (Na^+^, Ca^2+^), which is translated as an electrical signal. Reduced calcium (Ca^2+^) concentration activates guanylate cyclase activators, which promote the restoration of cGMP, thus acting as negative feedback. The termination of the visual response involves the phosphorylation of opsin by rhodopsin kinase (which is prevented by recoverin in the dark) and the binding of arrestin. In this study, we have identified genes coding for elements involved in the different stages of phototransduction as candidates for adaptive divergence between deepwater Malawi cichlids (*Diplotaxodon*), combining GWAS for eye size, haplotype-based selection scans, and differential gene expression analysis. These genes are in boxes colored according to the type of protein they encode. This illustration was inspired by Figure 2 of Yau and Hardie 2009, Figure 4 of Fain et al. 2010, and Figure 1 of Musilova et al. 2021.

Overall, rather than affecting eye size directly, we believe that many of the identified variants are causally linked to molecular traits of the visual system that co-vary with eye size, potentially because of concerted adaptation to specific light environments. Consistent with this, candidate genes showed broad enrichment for visual function. Previously, Malinsky et al. (2018) identified signals of parallel adaptation between *Diplotaxodon* and the deep benthic group involving genes in the cone phototransduction cascade, suggesting a key role of this pathway in-depth adaptation among Malawi cichlids. Our study builds on these findings by examining eye size-associated genetic variation within *Diplotaxodon*, allowing us to capture signals of adaptation occurring between species rather than across the entire clade. While we identified some of the same candidate genes (green-sensitive opsins, *arr3a*, *gnat2*, *grk7a*) (Malinsky et al. 2018), our analysis further revealed widespread signals of adaptation throughout the phototransduction cascade (Fig. 6). Furthermore, many of these genes showed differential expression between small and big-eyed species and/or signatures of selection. The cone arrestin *arr3a* stood out in our analysis as it harbored nonsynonymous mutations strongly associated with eye size variation, ranked among the most highly DE genes, and showed footprints of selection. Intriguingly, independent signals of selection in small and large-eyed species might point to diversifying selection—the main driver of sympatric speciation (Maynard 1962)—having acted on this gene, a hypothesis that warrants future research. Altogether, we find these results remarkable because they provide independent evidence for concerted adaptive evolutionary tinkering of the phototransduction pathway in the ecological divergence of *Diplotaxodon* species.

The phototransduction cascade is generally functionally conserved across vertebrates, and mutations in orthologs of genes identified in this study are associated with a large family of retinal diseases and vision impairments in humans (Felden et al. 2019; Solaki et al. 2022) and cone degeneration in animal models (Stearns et al. 2007; Naggert et al. 2023). Most of the phototransduction genes identified in our analysis are expressed in cones, which mediate vision in bright light conditions (Yokoyama 2008), and candidate nonsynonymous mutations are predominantly derived in big-eyed species. Together, this might suggest that some of these mutations disrupt functions not required in the depth range of big-eyed species, reflecting relaxed selection on cone function. However, the parallelism in the GWA signal, short evolutionary timescales, and signals of positive selection make it more likely that these mutations are adaptive. The fact that genes in the cone phototransduction cascade are evolving in parallel with the deep benthic clade, showing some of the same amino acid changes (Malinsky et al. 2018), further supports this. Nonsynonymous mutations could provide, for example, an energy-saving mechanism (Moran et al. 2015) or, more likely, confer a direct functional advantage, such as fine-tuning the speed of activation or shutdown of the visual pigment to match species-specific ecologies (e.g. Renninger et al. 2011; Morshedian et al. 2018). Aligning with this, we found strong expression differences in genes that collectively mediate the recovery of the visual pigment after light exposure, including recoverin (*rcvrn3*), rhodopsin kinase (*grk7a*), arrestin (*arr3a*) and guanylyl cyclase activators (*guca1b, guca1g*) (Figs. 4 and 6). We hypothesize that amino acid changes and expression differences in these genes could lead to altered cone response times, potentially mediating the trade-off between cone speed and sensitivity (Warrant and Locket 2004; Land and Nilsson 2012). For example, delayed cone responses in big-eyed *Diplotaxodon* could increase sensitivity to twilight (mesopic) conditions in their diurnal depth ranges or during nocturnal migrations to the surface (Thompson et al. 1996). It would be interesting to examine whether these species show other adaptations such as photoreceptor cell transmutations, as seen in mesopelagic deep-sea fishes (de Busserolles et al. 2017).

Our gene expression data indicate rod-dominated opsin expression in both small and big-eyed *Diplotaxodon* species, with cone opsin expression restricted to those sensitive to the blue-green region of the spectrum, consistent with patterns seen in organisms inhabiting dim habitats (reviewed in Carleton et al. 2020; de Busserolles et al. 2020; Musilova et al. 2021), including deepwater cichlids (Musilova, Indermaur et al. 2019; Ricci et al. 2023). Rhodopsin (*rh1*) mediates achromatic vision in dim-light conditions (Yokoyama 2008) and has been extensively implicated in deepwater adaptation (Sugawara et al. 2005; Nagai et al. 2011; Malinsky et al. 2015; Musilova, Cortesi et al. 2019; Ricci et al. 2022), also in *Diplotaxodon* (Hahn et al. 2017; Malinsky et al. 2018). The high rhodopsin expression observed could be driven by differences in photoreceptor abundance and influenced by eye size (Ricci et al. 2023). Further investigation, such as retinal microscopic examination and cell type-specific expression analyses (e.g. scRNAseq; Ogawa and Corbo 2021), is needed to determine whether this pattern reflects a higher number of rods in larger eyes or proportionally higher rod expression. Despite small and big-eyed species exhibiting typical “deepwater palettes”, there were substantial differences in the green opsin types expressed, with big-eyed *Diplotaxodon* almost exclusively expressing *rh2b* while small-eyed species expressed a combination of *rh2b* and *rh2aβ*. Visual modeling in the Malawi cichlid *Maylandia zebra* showed that co-expression of *rh2b* and *rh2aβ* can have a significant impact on contrast detection and color discrimination, as opposed to single expression of *rh2b* (Dalton et al. 2014). This suggests that differential opsin (co)expression in *Diplotaxodon* could provide species-specific ecological specializations. It would be interesting to examine how opsin expression varies within their retinas.

Overall, the limited opsin repertoire and consistently lower expression across several other (cone) phototransduction genes in big-eyed species suggest a decreased reliance on cone-based vision. However, the signatures of adaptation in these genes and expression of a cone green opsin, though at very low levels, suggest that color discrimination may be important in *Diplotaxodon*. In many deep-sea and nocturnal fish, green opsins are the only opsins remaining, though the functional relevance of expressing cone opsins in their extremely dim environments remains unclear (Musilova et al. 2021; Musilova and Cortesi 2023). In the deep, clear, open waters of Lake Malawi, predominant wavelengths align with peak absorbance of *rh2* genes (Carleton et al. 2016), possibly explaining the retention of cone function. This raises the question of whether opsin expression differences contribute to assortative-mating-based species isolation in our study system (Seehausen et al. 2008). *Diplotaxodon* species are expected to be fully interfertile. They coexist in an open-water environment void of any physical barriers and some genetically distinct but sympatric species are morphologically indistinguishable except by male breeding dress (Turner et al. 2004). Previous research has suggested a role for male nuptial patterning in species recognition and assortative mating in *Diplotaxodon* (Genner et al. 2007). Studying the link between species-specific opsin expression, spectral sensitivity, and the ability to discriminate nuptial patterning and hue in their light environments could offer meaningful insights into the role of visual adaptation in speciation. Unfortunately, their inaccessible habitat depths make it difficult to delimit their specific depth ranges and preclude behavioral studies.

Our analysis has also shed light on broader patterns of physiological depth adaptation, involving the hemoglobin complex—previously implicated in adaptation to anoxic and deepwater habitats in cichlids including *Diplotaxodon* (Hahn et al. 2017; Malinsky et al. 2018; Vernaz et al. 2022; Ricci et al. 2023)—and related genes with oxygen transport functions such as hepcidin and carbonic anhydrase. Interestingly, two of the candidate genes, *trhde* and *retsat*, have been linked to high-altitude adaptation in mammals, specifically in regulating body temperature and hypoxia response (Table 1; Edea et al. 2019; Xu et al. 2021). This highlights a certain degree of parallelism in selective pressures between deepwater and high-altitude environments, offering potential for comparative studies on parallel evolution. Besides this possible link to high-altitude adaptation, the presence of *trhde* among our candidates is intriguing because of its potential role in visual plasticity. This gene, which codes for thyrotropin-releasing hormone degrading peptide, is part of the thyroid hormone (TH) signaling pathway, known to influence spectral sensitivity in vertebrates by regulating opsin expression and chromophore shifts in visual pigments (Volkov et al. 2020). TH-mediated visual plasticity in response to light changes has been shown in Midas cichlids (Härer et al. 2017; Karagic et al. 2018, 2022) but its role remains underexplored in East African cichlids. It would be interesting to investigate whether *trhde* contributes to visual spectral tuning, potentially by modulating TH concentration in *Diplotaxodon* and, more generally, in cichlid radiations.

In conclusion, our study reveals that the evolution of eye size is tightly linked to broad visual adaptive tinkering largely involving the phototransduction cascade, uncovering some of the molecular mechanisms that have enabled *Diplotaxodon* to diversify in deepwater environments. While much previous research has focused on visual opsins due to their crucial role in modulating spectral sensitivity and their potential to aid speciation, further research is needed to elucidate the phenotypic and fitness impacts of the identified protein modifications and regulatory changes. Nonetheless, our results offer important insights into depth adaptation within one of the largest extant vertebrate evolutionary radiations, contributing to our understanding of how species radiate into novel environments.

## Materials and Methods

### Sampling and DNA Sequencing

A total of 1,360 samples were used in the analysis of eye size variation across the Malawi cichlid radiation and the *Diplotaxodon* genomics analyses (supplementary Data S1, Supplementary Material online). They were collected in 2016 and 2017 (with the exception of seven samples, which were collected in 2004) from fishermen along the shores of Lake Malawi, caught by a variety of methods including hook and line fishing, mid-water trawling, gill nets, and night-time Chirimila, a type of purse seine, or by the Ndunduma research trawler of the Fisheries Research Unit of the Government of Malawi. Specimens were photographed in left-hand side lateral view with a scale bar. Most of these samples have been included in previous studies (Malinsky et al. 2018; Svardal et al. 2020; Blumer et al. 2025). DNA was extracted from fin clips using standard protocols and short reads (100 to 150 bp paired-end) were obtained on Illumina HiSeq platforms as described in Malinsky et al. (2018) and Blumer et al. (2025).

For RNA sequencing, freshly caught, adult individuals were purchased from the Nkhata Bay market in 2018; their eyes immediately dissected after collection and preserved in 5 to 10 mL of RNA later. To minimize the potential influence of diurnal variation in gene expression, all dissections took place in the morning.

Details of the *Diplotaxodon* whole-genome sequences (*N* = 79) and the collection of samples for RNA sequencing (*N* = 10) included in this study can be found in supplementary Supplementary Data S3 and S4, Supplementary Material online. Sample collection, export, and use are covered by permits of the Government of Malawi.

### Alignment and Variant Calling

Sequencing reads were aligned to the high-quality reference genome of a closely related Malawi cichlid, *Astatotilapia calliptera* (<0.3% sequence divergence, fAstCal1.2, GCA_900246225.3) using the mem algorithm of bwa v0.7.17. Variants were called and filtered using bcftools v1.9. Sites were excluded if they showed poor read mapping, unusual depth of coverage, biased allele balance in heterozygotes, or an excess of missing genotypes. Specifically, sites with a root mean square MQ below 50.0 and with a fraction of MQ zero reads greater than 0.10 were filtered to remove low-confidence calls. Sites were further excluded if more than 20% of the genotypes were missing, if the test of excess heterozygosity as implemented in bcftools showed a value below 0.2, if the Mann–Whitney *U* test of MQ versus strand bias was highly significant (*P* < 0.001), or if allele balance in heterozygous calls was significantly skewed (*P* < 0.01). Additionally, sites with abnormally variable read depths were excluded using a depth deviation metric, defined as the average across all samples of the deviation of read depth at a focal site from the genome-wide mean coverage of each individual, expressed in units of standard deviation. Sites with a depth deviation > 1.5 were filtered out. Biallelic SNPs passing all filtering criteria were phased using Eagle v2.4.1 (Loh et al. 2016) with “--Kpbwt = 40,000” and “--expectIBDcM = 0.5” to suit the cichlid model (Blumer et al. 2025). Missing genotypes were imputed (default). The variant calling workflow was performed on a larger sample set of 1,465 Lake Malawi cichlids, most of which are presented in Blumer et al. (2025), and then down sampled to only include the *Diplotaxodon* individuals used in this study (supplementary Data S3, Supplementary Material online).

### *Diplotaxodon* Genetic Relationships

Pairwise genetic differences were calculated using custom Python code available at https://github.com/feilchenfeldt/pypopgen3. A neighbor-joining (NJ) tree was constructed using scikit-bio v0.5.6. The tree was rooted using an ancestral sequence reconstructed from whole-genome alignments between the *A. calliptera* reference genome and outgroup species (data and methods available in Blumer et al. 2025).

### Eye Size and Body Shape Measurements

Morphological data were obtained from coordinates of 18 homologous landmark points from digital photographs following Theis et al. (2014) (see supplementary fig. S1, Supplementary Material online therein), using tpsDig2 v2.26 (Rohlf 2004). The landmarks surrounding the eye were used for the eye size analysis. To remove the effect of body size on eye size variation, for the GWA analysis we defined eye size as the ratio of horizontal eye diameter to standard length (supplementary fig. S7, Supplementary Material online). Eye size measurements were obtained for 75 *Diplotaxodon* specimens and for 1,259 specimens of the remaining five eco-morphological clades of the radiation included in this study (supplementary Data S1 and S2, Supplementary Material online).

A general measure of body shape, used in the calculation of the correlation between *Diplotaxodon* body shape differences and genetic distance (supplementary fig. S3, Supplementary Material online), was obtained by summarizing gross body morphology from coordinates of all 18 landmarks (supplementary fig. S7, Supplementary Material online) using principal component analysis (PCA) in MorphoJ v1.07a. To remove variation due to size, orientation, and position of the fish, we first applied a Procrustes superimposition of the data. Subsequently, PCA was applied to the Procrustes coordinates to summarize shape variation. The first principal component (PC1), accounting for 54% of the variation, was used as a representative measure of body shape.

### Phylogenetic Signal

As a measure of phylogenetic signal for eye size in *Diplotaxodon*, we estimated Pagel's lambda (λ) using the R package phytools (Revell 2012) v.0.7.70 (function “phylosig”).

### GWA Analysis

The GWA analysis for eye size was conducted by fitting a univariate linear mixed model using the software GEMMA v0.98 (Zhou and Stephens 2012). Sites with minor allele frequency (MAF) < 5% were excluded. A centered relatedness matrix (option “-gk 1”) was used to account for population structure. After MAF filtering, 1,900,366 SNPs were analyzed. The *P*-values reported correspond to the likelihood ratio test computed by GEMMA. A total of 53 specimens of 9 *Diplotaxodon* species were included in this analysis. These were all specimens for which both phenotypes and sequences were available (supplementary Data S3, Supplementary Material online). Note that sample sizes differed between species (see supplementary table S1, Supplementary Material online).

### Principal Component Analysis

PCAs to summarize genetic variation in GWA outliers (top-ranking 0.01% SNPs, *N* = 190) and genome-wide were conducted using PLINK v1.9. For the PCA of genome-wide variation, SNPs with MAF < 0.05 were excluded using bcftools v1.14 “--min-af 0.05:minor” and SNPs were linkage-disequilibrium pruned using the option “--indep-pairwise 50 10 0.1” in PLINK. The genome-wide PCA was performed on the remaining 377,273 SNPs. The linear regression model of PC1 on eye size was fitted by ordinary least squares (OLS) using the Python module statsmodels v0.14.0 (supplementary table S2, Supplementary Material online).

### Identification of GWA Outliers and Candidate Genes

GWA outliers were identified based on the top 0.01% most significant associations (190 SNPs, *P* < 0.0001), corresponding to the 99.99th percentile of the −log_10_(*P*-value) distribution. This threshold was chosen to increase the number of variants for downstream analyses while retaining the strongest signals. Additional thresholds, such as the 0.025% outlier level, and exploratory searches for vision-related genes were conducted to provide broader context but all downstream analyses were based on the SNPs passing the 0.01% cutoff, referred to as GWA outliers throughout the text. Candidate genes were identified as those containing nonsynonymous SNPs among the GWA outliers (17 genes, Table 1).

### Annotation of GWA Outlier SNPs

The SNP annotation was performed using snpEff v5.1 (Cingolani, Platts, et al. 2012) using a prebuilt database for the *Astatotilapia calliptera* reference genome fAstCal1.2 (GCA_900246225.3; GenBank assembly). Throughout the text, genes are referred to as annotated by snpEff (supplementary Data S5, Supplementary Material online). Whenever a gene name/symbol was missing but an Ensembl gene identifier (ENSACLG) was present instead, we manually searched for a match in the RefSeq genome assembly GCF_900246225.1 using the NCBI gene database (https://www.ncbi.nlm.nih.gov/gene/) and the NCBI Genome Data Viewer (https://www.ncbi.nlm.nih.gov/genome/gdv/browser/genome/?id=GCF_900246225.1).

### GO Enrichment Analysis

The GO enrichment analysis was performed using the R package topGO v2.38.1 (Alexa and Rahnenfuhrer 2019) from the Bioconductor project (www.bioconductor.org).

As test gene set, we included all genes with transcription start sites (TSS) located within ± 25 kb of a GWA outlier SNP. This window was chosen to capture genes directly harboring outlier variants in their gene bodies, as well as those that may be cis-regulated by nearby variants due to their proximity to target promoters (Kleinjan and van Heyningen 2005). The background gene set was generated by extracting the protein-coding genes from the annotated GWA callset using the SnpSift (Cingolani, Patel et al. 2012) “extractFields” option. To map the genes to GO terms, we used the zebrafish annotation package (org.Dr.eg.db) from Bioconductor. Prior to this, Ensembl gene identifiers had been converted to their “gene symbol”, when possible, to maximize the number of genes that were mapped to GO terms, using the Ensembl BioMart data mining tool (Ensembl Release 108, fAstCal1.2 genes) and g:Convert, provided by the web server g:Profiler (Raudvere et al. 2019). Despite implementing this approach, some genes lacked correspondence to zebrafish genes (e.g. paralogs of duplicated genes) and were thus not included in the analysis. To check for potentially relevant genes that could have been excluded this way, we manually inspected the annotation of GWA outliers for *Danio rerio* genes in the GO terms by browsing the AmiGO 2 database (https://amigo.geneontology.org/amigo/landing).

A total of 11,623 feasible genes could be mapped to terms in the Biological Process (“BP”) ontology. The minimum number of genes mapping to a GO category was set to 5 (option “nodeSize = 5”). Statistical overrepresentation of GO terms was calculated by performing Fisher's exact test, using the weight algorithm (object class “weightCount”). This method improves explanatory power over the classic GO scoring algorithm by taking into account the local dependencies between GO terms that arise as a consequence of the hierarchical structure of the GO graph (Alexa et al. 2006). The estimated *P*-values (supplementary table S3, Supplementary Material online) are reported without further multiple testing correction, as suggested by the authors (Alexa and Rahnenfuhrer 2019).

### Testing for Overrepresentation of Functional Annotations Among GWA Outliers

To test whether any of the functional effects predicted by snpEff was overrepresented in the genomic annotation of GWA outliers, we compared the observed number of GWA outliers per annotation effect to an empirical distribution obtained by 1,000 draws of randomized sets of SNPs, matched on MAF, as described in the following.

First, using bcftools v1.14, we extracted MAF per position for the entire callset used in the GWAS (control set) and in a reduced subset of this containing only the GWA outliers (test set, 190 SNPs). SNPs in the control set were grouped into discrete nonoverlapping MAF bins of width 0.05. Then, in each iteration, a set of 190 random SNPs matching the test set on MAF distribution was drawn from the control set. The number of SNPs per annotation effect in each random set of SNPs was counted to generate a genome-wide distribution of effects (note that one SNP can have more than one annotation). The empirical *P*-value was calculated as the proportion of permutations in which the number of SNPs with an annotation effect was larger than the observed number in the test set (supplementary fig. S4, Supplementary Material online).

The functional annotation categories tested for overrepresentation were: “3-prime UTR variant”, “Downstream gene variant”, “Upstream gene variant”, “Intergenic region”, “Intron variant”, “Synonymous variant”, “Missense variant” and “Splice region variant and intron variant”.

### Patterns of Allelic Segregation at Nonsynonymous GWA Outliers

To gain an overview of which alleles at nonsynonymous GWA outlier sites might have been under selection in small and big-eyed *Diplotaxodon* species, we inspected allelic segregation of each outlier across the phylogeny. For this analysis, we used a larger data set with all sequenced samples (*N* = 79), including those for which no eye size data was available and that had thus not been included in the GWAS (supplementary Data S3, Supplementary Material online).

The data was phased so that the reference allele corresponded to the ancestral state and the alternative allele to the derived state (see “Alignment and variant calling”). For each nonsynonymous site, we calculated allele frequency (AF) per species when at least two samples were available. Then, species for which the derived allele was dominant (AF > 0.5) at each site were grouped based on whether the group consisted of small-eyed species or big-eyed species, assigning *D. apogon*, *D. macrops* and D. “bigeye black dorsal” as big-eyed species and *D. limnothrissa*, *D.* “limnothrissa black dorsal”, *D.* “holochromis 1” and *D.* “holochromis 2” as small-eyed species (supplementary table S4, Supplementary Material online). We observed that while most species consistently clustered within their eye size group, *D. longimaxilla* and *D.* “limnothrissa black dorsal” were found within both groups, not following a clear pattern, and were therefore ignored. We counted the number of sites for which the derived allele was dominant in each group. Following the observation that the derived allele was almost consistently dominant or fixed in the big-eyed species group for the phototransduction genes (11 out of 12 sites; supplementary table S4, Supplementary Material online), we tested whether this pattern of segregation was not independent of the species group by performing a chi-square test. This was repeated for genes with putative skeletal muscle functions.

### Signals of Selection in Candidate Genes

To detect signatures of selection around candidate genes, i.e. genes annotated to nonsynonymous GWA outliers, we applied two haplotype-based tests: the cross-population extended haplotype homozygosity statistic, XP-EHH (Sabeti et al. 2007), and the iHS (Voight et al. 2006). Both statistics are based on the EHH concept (Sabeti et al. 2002), which measures the length of a haplotype around a focal SNP, and work best for relatively recent sweeps, before the long, selected haplotypes are broken down by recombination. The tests differ in their power to detect complete or partial sweeps. Specifically, XP-EHH compares EHH between two populations and is used to identify loci under divergent selection, in which the selected haplotype is near or has reached fixation in one population but remains polymorphic in the other. iHS assesses EHH within a population and is used to detect very recent or partial selective sweeps, which appear as large differences in haplotype length of the ancestral and derived allele at the locus under selection. This analysis was performed using the R package rehh v3.2.2 (Gautier et al. 2017) on the two species with largest sample sizes representative of “small” and “large” eye *Diplotaxodon*: *D. limnothrissa* (*N* = 33) and *D.* “bigeye black dorsal” (*N* = 20), respectively.

We computed XP-EHH along the genome and assessed whether XP-EHH scores around the candidate genes represent outliers in the genome-wide distribution of XP-EHH scores. To do this, we computed the distribution of maximum XP-EHH scores (most positive or negative values) in 10,000 randomly selected 30 kb windows along the genome. Based on this distribution, we then ranked the maximum XP-EHH scores in 30 kb windows centered around nonsynonymous GWA outliers. Windows with top-ranking XP-EHH scores (quantile > 0.95 or < 0.05) were considered to have been under recent divergent selection.

To search for additional signals of recent selective sweeps within each population (*D. limnothrissa* and *D*. “bigeye black dorsal”), we computed iHS along the genome using rehh v3.2.270 with default parameters. As we were interested in selection on sites contributing to the divergence between small and big-eyed species, we visually inspected the candidate regions (defined as above) for peaks of absolute iHS scores present for one species but not the other.

### Coverage of Mapped Reads Around arr3a Locus

To check for a potential duplication of the gene *arr3a*, we inspected the coverage of reads mapped to the reference genome using samtools v1.14 (Li et al. 2009). We estimated the median coverage in the *arr3a* gene region (chr10:19,813,620–19,820,182) per sample and normalized it by the median genome-wide coverage.

### RNA Extraction, Library Preparation and Sequencing

All procedures were conducted on ice unless otherwise specified. Eye samples stored in RNAlater were thawed from −80 °C and transferred to 5 mL of Trizol. Each sample was then homogenized using a handheld homogenizer and 1 mL of the homogenate was chilled in Trizol and allowed to rest for 5 min. Next, 200 μL of chloroform (ThermoFisher Scientific) was added and the samples were vigorously shaken for 15 s, briefly vortexed, and incubated at room temperature for 15 min. The samples were then centrifuged at 300*×g* for 20 min at 4 °C. The supernatant was carefully transferred to a fresh tube and further processed using the Direct-Zol RNA Purification Kit (Zymo) according to the manufacturer's instructions. The quality and quantity of the extracted total RNA were assessed using Qubit (RNA BR assay, Agilent) and Tapestation (Agilent). Total RNA extracted from each eye was submitted individually for sequencing. All libraries were prepared, quality-controlled, and sequenced by Novogene Corporation (China) using the Illumina NovaSeq 6000 platform to generate paired-end reads of 150 base pairs (bp). Raw sequencing reads have been deposited in the Sequence Read Archive (SRA) under BioProject accession number PRJNA1271605 (supplementary Data S4, Supplementary Material online).

### Differential Gene Expression Analysis

The raw reads from RNA-seq were mapped against the *Astatotilapia calliptera* reference genome (NCBI RefSeq accession GCF_900246225.1) using STAR v2.7.10b (Dobin et al. 2013) and the number of read counts within gene coding sequences (CDS) summarized using htseq-count v2.0.4 (Anders et al. 2015). Differential expression analysis was conducted using the R package DESeq2 v1.42.0 (Love et al. 2014). Before the analysis, the read count table was filtered to retain only protein-coding genes, and genes with low read counts were excluded by requiring at least five counts in a minimum of three samples. After filtering, 20,299 genes out of 26,070 protein-coding genes in the count dataset were included in the differential expression analysis. DE genes between the *D. limnothrissa* and *D.* “macrops black dorsal/offshore” species groups were identified as those with a Wald test (default) adjusted *P*-value (Benjamini–Hochberg method) below the cutoff FDR < 0.05. To facilitate the ranking of DE genes by the log2 fold change, we applied the apeglm method (Zhu et al. 2019) for effect size shrinkage.

### Opsin Gene Expression

To examine the expression profile of visual opsin genes, we normalized the raw read counts outputted by htseq-count (see above) as transcripts per million (TPM) and identified opsin genes by searching for orthologs to the *Oreochromis niloticus* (supplementary table S6, Supplementary Material online). Using the normalized values, we calculated cone and rod opsin expression relative to total opsin gene expression and individual cone opsin expression relative to total cone opsin expression. Due to the high degree of sequence similarity between the two copies of *RH2A*, i.e. *RH2Aα* and *RH2Aβ*, (> 90%) and given that the purpose of this analysis was to obtain a general overview rather than an in-depth characterization opsin expression patterns, the expression values of reads mapping to either copy were summed up and collectively expressed as *RH2A*.

## Supplementary Material

msaf147_Supplementary_Data

## Data Availability

Raw whole-genome sequencing reads for *Diplotaxodon* samples published in previous studies are available in the European Nucleotide Archive (ENA) under BioProjects PRJEB1254, PRJEB15289, and PRJEB21343. RNA-seq reads are available under BioProject PRJNA1271605. Corresponding BioSample accessions are listed in supplementary Data S3 and S4, Supplementary Material online. Variant call format (VCF) files are available on Zenodo: https://doi.org/10.5281/zenodo.15576996. The code and additional data associated with this study are available on GitHub (https://github.com/j-camacho/Diplotaxodon_deepwater_adaptation) and archived at Zenodo: https://doi.org/10.5281/zenodo.15602855. We note that the genetic material and sequences used in this study are subject to an Access and Benefit Sharing (ABS) agreement with the Government of Malawi. Any person who wishes to use this data for any form of commercial purpose must first enter into a commercial licensing and benefit-sharing agreement with the government of Malawi.
