# Supplementary material for: Widespread Genetic Signals of Visual System Adaptation in Deepwater Cichlid Fishes
Source: Mol Biol Evol. 2025 Jun 9;42(7):msaf147. doi: 10.1093/molbev/msaf147 (PMC12210959; doi:10.1093/molbev/msaf147)
Supplement: msaf147_Supplementary_Data [file msaf147_supplementary_data.zip › diplotaxodon_adaptation_supplement_submission.pdf]

Table of Contents

Supplementary tables 2

Table S1. Summary of *Diplotaxodon* samples used in the different analyses of this study 2

Table S2. Linear regression results of relative eye size on PC1 from GWA outlier SNPs and genome-wide variation. 3

Table S3. Gene ontology (GO) enrichment test results of GWA outliers 4

Table S4. Summary of allele frequency distribution per species group at nonsynonymous GWA outliers 6

Table S5. Top 40 differentially expressed genes across whole-eye transcriptomes of small-eyed and big-eyed *Diplotaxodon* 7

Table S6. Visual opsin genes annotated in the *A. calliptera* reference genome (GCF_900246225.1).......... 8

Supplementary figures 9

Figure S1. Eye size variation in Lake Malawi cichlids 9

Figure S2. *Diplotaxodon* samples phylogeny 10

Figure S3. Eye size (a) and body shape (b) differences plotted against pairwise genetic distance in *Diplotaxodon*.......... 11

Figure S4. Empirical distributions of SNP annotations from 1,000 random draws of SNPs 12

Figure S5. Median coverage per sample across the *arr3a* (LOC113031132) gene region.......... 13

Figure S6. Normalized cone opsin expression (transcripts per million, TPM) 14

Figure S7. Homologous landmark points used in this study. 15

Supplementary tables

Table S1. Summary of *Diplotaxodon* samples used in the different analyses of this study.

**Species**	Whole-genome sequences	Phenotyped	GWAS	Allele frequency nonsynonymous GWA outliers	Selection (XPEHH, iHS)	RNA-seq, gene expression
D. apogon	**2**	**3**	**2**	**2**	0	0
D. ecclesi	**1**	**1**	**1**	0	0	0
D. limnothrissa	**33**	**27**	**16**	**33**	**33**	**5**
D. longimaxilla	**6**	**3**	**3**	**6**	0	0
D. macrops	**8**	**7**	**7**	**8**	0	0
D. sp. 'bigeye black dorsal'	**20**	**20**	**19**	**20**	**20**	
D. sp. 'holochromis'	**2**	**8**	**2**	**2**	0	0
D. sp. 'limnothrissa black dorsal'	**2**	**2**	**2**	**2**	0	0
D. sp. 'limnothrissa black pelvic'	**1**	**1**	**1**	0	0	0
D. greenwoodi	**1**	**3**	0	0	0	0
D. sp. 'bigeye white dorsal'	**1**	0	0	0		0
D. sp. 'ngulube'	**1**	0	0	0	0	0
D. cf. macrops 'black dorsal'	0	0	0	0	0	**3**
D. cf. macrops 'offshore'	0	0	0	0	0	**2**
Pallidochromis tokolosh	**1**	0	0	0	0	0
Total	79	75	53	73	53	10

Table S2. Linear regression results of relative eye size on PC1 from GWA outlier SNPs and genome-wide variation. Top: Principal Component 1 (PC1) based on outlier SNPs (top 0.01% most significant, 190 SNPs) from a genome-wide association (GWA) study for relative eye size across 9 *Diplotaxodon* species (N = 53); Bottom: PC1 derived from genome-wide variation (377,273 SNPs).

```
                            OLS Regression Results                            
==============================================================================
Dep. Variable:                  ED.SL   R-squared:                       0.831
Model:                            OLS   Adj. R-squared:                  0.828
Method:                 Least Squares   F-statistic:                     251.5
Date:                Tue, 12 Sep 2023   Prob (F-statistic):           2.34e-21
Time:                        20:07:54   Log-Likelihood:                 193.60
No. Observations:                  53   AIC:                            -383.2
Df Residuals:                      51   BIC:                            -379.3
Df Model:                           1                                         
Covariance Type:            nonrobust                                         
==============================================================================
                 coef    std err          t      P>|t|      [0.025      0.975]
------------------------------------------------------------------------------
const          0.0980      0.001    111.605      0.000       0.096       0.100
PC1           -0.1014      0.006    -15.858      0.000      -0.114      -0.089
==============================================================================
Omnibus:                        0.254   Durbin-Watson:                   1.364
Prob(Omnibus):                  0.881   Jarque-Bera (JB):                0.437
Skew:                           0.106   Prob(JB):                        0.804
Kurtosis:                       2.609   Cond. No.                         7.28
==============================================================================
```

```
                            OLS Regression Results                            
==============================================================================
Dep. Variable:                  ED.SL   R-squared:                       0.470
Model:                            OLS   Adj. R-squared:                  0.459
Method:                 Least Squares   F-statistic:                     45.18
Date:                Wed, 13 Sep 2023   Prob (F-statistic):           1.50e-08
Time:                        15:44:38   Log-Likelihood:                 163.24
No. Observations:                  53   AIC:                            -322.5
Df Residuals:                      51   BIC:                            -318.5
Df Model:                           1                                         
Covariance Type:            nonrobust                                         
==============================================================================
                 coef    std err          t      P>|t|      [0.025      0.975]
------------------------------------------------------------------------------
const          0.0980      0.002     62.932      0.000       0.095       0.101
PC1           -0.0762      0.011     -6.721      0.000      -0.099      -0.053
==============================================================================
Omnibus:                        0.984   Durbin-Watson:                   0.881
Prob(Omnibus):                  0.611   Jarque-Bera (JB):                1.016
Skew:                           0.212   Prob(JB):                        0.602
Kurtosis:                       2.470   Cond. No.                         7.28
==============================================================================

Notes:
[1] Standard Errors assume that the covariance matrix of the errors is co
rrectly specified.
```

Table S3. Gene ontology (GO) enrichment test results of GWA outliers. GO terms significant at *P* < 0.05 (Fisher's exact test) from the topGO 'weight' algorithm and their ranking in the same test performed using the 'classic' algorithm are shown. The list of annotated *Astatolilapia calliptera* (fAstCal1.2.99) genes corresponding to each GO term is included.

GO.ID	Term	Annotated	Found	Expected	Rank in classic	P-value (weight)	Genes
GO:0036368	cone photoresponse recovery	6	2	0.04	2	0.00062	*grk7a, rcvrn3*
GO:0009584	detection of visible light	12	2	0.08	5	0.00267	*gnat2, grk7a*
GO:0007601	visual perception	100	4	0.65	6	0.00412	*arr3a, cnga3a, gnat2, grk7a*
GO:0010499	proteasomal ubiquitin-independent protein catabolic process	19	2	0.12	9	0.00671	*psma6b, psmb6*
GO:0003197	endocardial cushion development	5	1	0.03	18	0.03227	*mmd*
GO:0007525	somatic muscle development	5	1	0.03	19	0.03227	*sgcd*
GO:0007612	learning	5	1	0.03	20	0.03227	*pappaa*
GO:0019835	cytolysis	5	1	0.03	21	0.03227	*mmd*
GO:0030719	P granule organization	5	1	0.03	22	0.03227	*pld6*
GO:0035066	positive regulation of histone acetylation	5	1	0.03	23	0.03227	*tada2b*
GO:0042539	hypotonic salinity response	5	1	0.03	24	0.03227	*cahz*
GO:0043697	cell dedifferentiation	5	1	0.03	25	0.03227	*ascl1a*
GO:0048798	swim bladder inflation	5	1	0.03	26	0.03227	*sgcd*
GO:0060575	intestinal epithelial cell differentiation	5	1	0.03	27	0.03227	*ascl1a*
GO:0072132	mesenchyme morphogenesis	5	1	0.03	28	0.03227	*mmd*
GO:0072425	signal transduction involved in G2 DNA damage checkpoint	5	1	0.03	29	0.03227	*babam1*
GO:0090140	regulation of mitochondrial fission	5	1	0.03	30	0.03227	*mief2*
GO:1902116	negative regulation of organelle assembly	5	1	0.03	31	0.03227	*smcr8b*
GO:2000785	regulation of autophagosome assembly	5	1	0.03	32	0.03227	*smcr8b*
GO:0002031	G protein-coupled receptor internalization	6	1	0.04	53	0.03860	*arr3a*
GO:0006729	tetrahydrobiopterin biosynthetic process	6	1	0.04	54	0.03860	*gch1*
GO:0014028	notochord formation	6	1	0.04	55	0.03860	*pacsin3*
GO:0019755	one-carbon compound transport	6	1	0.04	56	0.03860	*cahz*
GO:0043046	DNA methylation involved in gamete generation	6	1	0.04	57	0.03860	*pld6*

GO:0043114	regulation of vascular permeability	6	1	0.04	58	0.03860	*pawr*
GO:0046654	tetrahydrofolate biosynthetic process	6	1	0.04	59	0.03860	*gch1*
GO:0048566	embryonic digestive tract development	6	1	0.04	60	0.03860	*ascl1a*
GO:0097150	neuronal stem cell population maintenance	6	1	0.04	61	0.03860	*fezf2*
GO:0016239	positive regulation of macroautophagy	7	1	0.05	70	0.04489	*smcr8b*
GO:0060012	synaptic transmission, glycinergic	7	1	0.05	71	0.04489	*glra4b*

Table S4. Summary of allele frequency distribution per species group at nonsynonymous GWA outliers. Per SNP, it is noted whether the derived allele is at high frequency (AF > 0.5) in big-eyed or small-eyed species. Grouping: ‘Bigeye’ = *D. macrops*, *D. apogon* and *D.* ‘bigeye black dorsal’; ‘Smalleye’ = *D. limnothrissa*, *D.* ‘limnothrissa black dorsal’, *D.* ‘holochromis 1’ and *D.* ‘holochromis 2’. Genes mapping to each SNP are specified as annotated by snpEff. For ensembl gene IDs, NCBI gene names are given if available. Phototransduction genes are highlighted in bold.

Position	snpEff gene (GenBank)	RefSeq gene	Species group AF>0.5
chr4:3475861	*hlfa*		Smalleye
chr4:16957197	*pvalb1*		Smalleye
chr4:16960791	*pvalb1*		Smalleye
chr4:16960806	*pvalb1*		Smalleye
chr5:8177566	ENSACLG00000000512[1]	-	Smalleye
chr5:8177868	ENSACLG00000000512	-	Smalleye
chr5:8177944	ENSACLG00000000512	-	Smalleye
chr5:8178415	ENSACLG00000000512	-	Smalleye
chr5:8179344	ENSACLG00000000512	-	Smalleye
chr5:8179371	ENSACLG00000000512	-	Smalleye
chr16:803056	ENSACLG00000007199[2]	-	Smalleye
chr16:8224701	ENSACLG00000019534	*neb*	Smalleye
chr16:8226565	ENSACLG00000019534	*neb*	Smalleye
chr16:8226567	ENSACLG00000019534	*neb*	Smalleye
chr18:34668946	*pappa2*		Smalleye
chr23:12485802	***cnga3a***		Smalleye
chr4:25465376	**ENSACLG00000017357**	LOC113021214 (*pde6h*-like)	Bigeye
chr4:25465378	**ENSACLG00000017357**	LOC113021214	Bigeye
chr5:37739275	ENSACLG00000000072	*camk1ga*	Bigeye
chr7:36736953	ENSACLG00000005135	*crata*	Bigeye
chr8:7058216	si:ch1073-13h15.3		Bigeye
chr8:7060038	si:ch1073-13h15.3		Bigeye
chr8:15826466	ENSACLG00000015226	LOC113028588 (parvalbumin β)	Bigeye
chr10:12393763	***gucy2d***		Bigeye
chr10:12394689	***gucy2d***		Bigeye
chr10:19815931	***arr3a***		Bigeye
chr10:19816225	***arr3a***		Bigeye
chr17:18162344	*trhde.2*		Bigeye
chr18:35018105	*cahz*		Bigeye
chr20:27271911	***gnat2***		Bigeye
chr23:12485592	***cnga3a***		Bigeye
chr23:12485741	***cnga3a***		Bigeye
chr23:12485800	***cnga3a***		Bigeye
chr23:12486012	***cnga3a***		Bigeye

[1]SNP overlapping LOC113022044 (*igfn1*); [2]SNP overlapping LOC113007658 (*ttn*).

Table S5. Top 40 differentially expressed genes across whole-eye transcriptomes of small-eyed and big-eyed *Diplotaxodon*. The log2 fold change (‘Log2FC’) was calculated for small-eyed vs. big-eyed (log2(small-eyed/big-eyed)) and the values reported are after applying log fold change shrinkage using the apeglm method in DESeq2. Vision-related genes are highlighted in bold. ‘Small-eyed’: *D*. ‘limnothrissa complex’, N = 5; ‘Big-eyed’: *D.* cf. ‘macrops black dorsal’ and *D.* cf. ‘macrops offshore’, N= 5.

Gene symbol	Gene ID	Gene name	Log2FC
Overexpressed in *D. limnothrissa* relative to *D*. ‘macrops black dorsal/offshore’			
	**LOC113022256**	**green-sensitive opsin-like**	**11.48**
	LOC113020500	hemoglobin subunit beta-A	9.53
**guca1g**	**LOC113037255**	**guanylyl cyclase-activating protein 2-like**	**9.20**
	LOC113020505	hemoglobin subunit alpha-A-like	9.00
	**LOC113022253**	**green-sensitive opsin-like**	**7.83**
**arr3a**	**LOC113031132**	**arrestin-C-like**	**7.80**
	LOC113026102	lysozyme C-like	7.40
cnga4	LOC113007954	cyclic nucleotide gated channel subunit alpha 4	7.26
	LOC113016003	inosine-uridine preferring nucleoside hydrolase-like	6.45
**pde6ha**	**LOC113024089**	**retinal cone rhodopsin-sensitive cGMP 3',5'-cyclic phosphodiesterase subunit gamma-like**	**6.28**
**guca1b**	**LOC113016882**	**guanylate cyclase activator 1B**	**6.21**
	LOC113020510	hemoglobin subunit beta-like	6.15
	LOC113014030	TELO2-interacting protein 1 homolog	5.79
si:ch73-141c7.1	LOC113019992	coenzyme Q-binding protein COQ10 homolog, mitochondrial	5.78
**grk7**	**LOC113010589**	**G protein-coupled receptor kinase 7a**	**5.72**
	LOC113011215	C-C motif chemokine 18-like	5.72
	LOC113023841	GTPase IMAP family member 7-like	5.62
**rcvrn3**	**LOC113015211**	**recoverin-like**	**5.55**
	LOC113006406	selenoprotein H-like	5.45
hbz	LOC 113020501	hemoglobin subunit zeta	5.43
Underexpressed in *D. limnothrissa* relative to *D*. ‘macrops black dorsal/offshore’			
	LOC113032212	hepcidin-like	-11.30
	LOC113032605	hepcidin-like	-8.23
	LOC113012651	uncharacterized	-7.56
	LOC113032603	hepcidin-like	-7.44
	LOC113030714	collagenase 3-like	-6.87
b3galt8	LOC113034269	beta-1,3-galactosyltransferase 8	-6.36
hbbe3	LOC113028620	hemoglobin subunit beta-1-like	-6.05
	LOC113027345	heat shock protein 30-like	-5.98
hbae5	LOC113028621	hemoglobin subunit alpha-like	-5.91
	LOC113020897	uncharacterized	-5.58
MFAP4	LOC113028121	microfibril-associated glycoprotein 4-like	-5.35
	LOC113020516	hemoglobin embryonic subunit alpha-like	-5.03
	LOC113033737	interleukin-1 beta-like	-5.02
	LOC113013774	coiled-coil domain-containing protein 80-like	-5.02
	LOC113015684	hemicentin-2-like	-4.97
	LOC113025244	homeodomain-interacting protein kinase 3-like	-4.91
acod1	LOC 113008072	aconitate decarboxylase 1	-4.82
arx	LOC113029758	aristaless related homeobox a	-4.79
	LOC113032734	IgGFc-binding protein-like	-4.70
	LOC113020595	myosin heavy chain, fast skeletal muscle-like	-4.55

Table S6. Visual opsin genes annotated in the *A. calliptera* reference genome (GCF_900246225.1).

Opsin	Gene ID	Gene name	Scaffold	Start	End	Gene length
RH1	LOC113013042	rhodopsin	NC_039321.1	8359611	8361117	1507
RH2B	LOC113022254	green-sensitive opsin	NC_039306.1	15163687	15167069	3383
RH2Aα	LOC113022253	green-sensitive opsin-like	NC_039306.1	15173872	15175937	2066
RH2Aβ	LOC113022256	green-sensitive opsin-like	NC_039306.1	15185496	15187269	1774
SWS1	LOC113009129	opsin 1, short-wave-sensitive 1 (opn1sw1)	NC_039318.1	15517458	15519123	1666
SWS2A	LOC113022898	blue-sensitive opsin-like	NC_039306.1	30059325	30061723	2399
SWS2B	LOC113022899	blue-sensitive opsin	NC_039306.1	30066006	30069025	3020
LWS	LOC113022900	red-sensitive opsin	NC_039306.1	30074467	30076799	2333

Supplementary figures

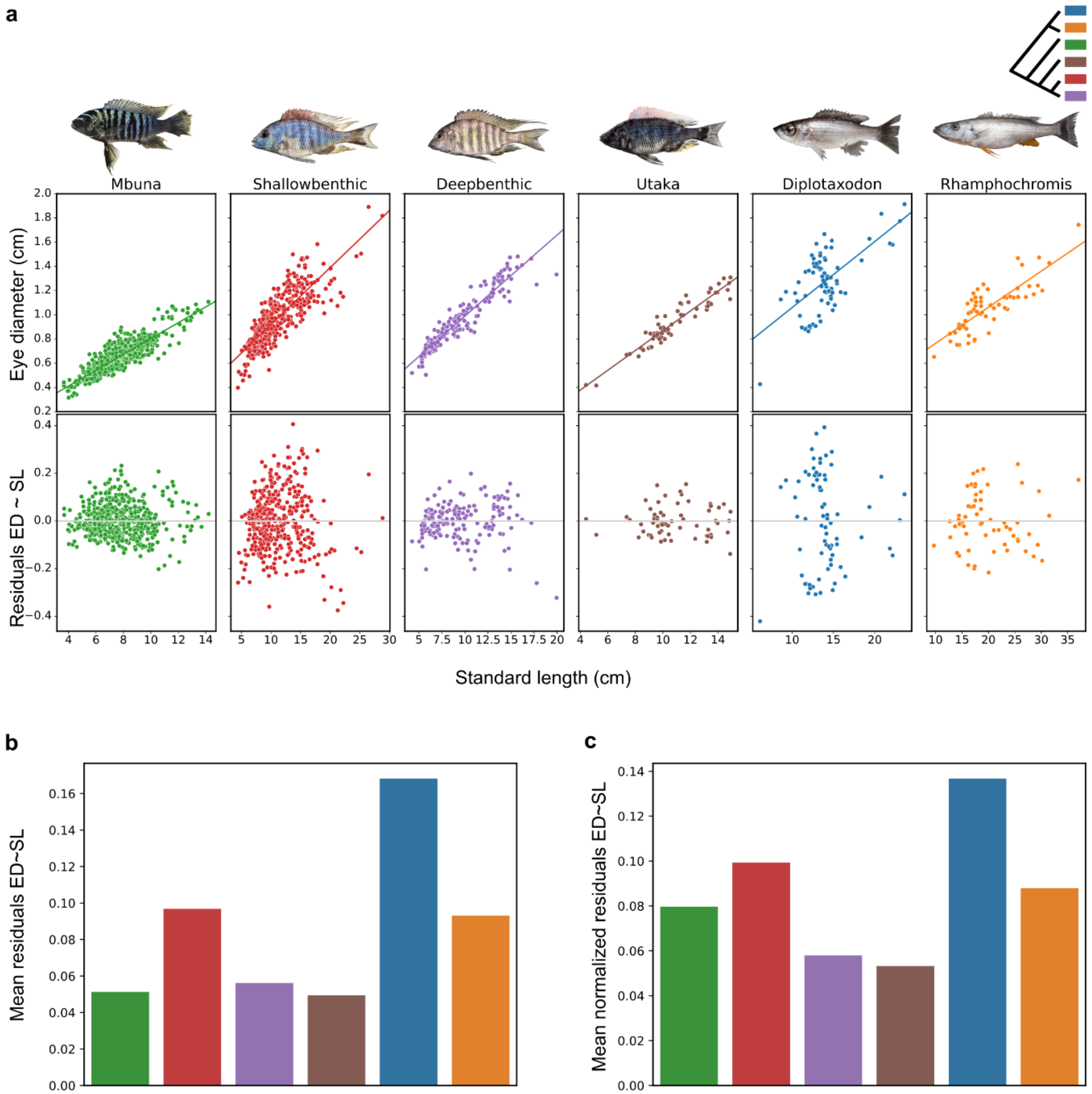

Figure S1. Eye size variation in Lake Malawi cichlids. **a)** Top: Linear regressions of eye horizontal diameter against standard length in the main ecomorphological clades of the radiation (Mbuna, N = 563; Shallow benthic, N = 401; Deep benthic, N = 170; Utaka, N = 63; Diplotaxodon, N = 75; Rhamphochromis, N = 62); Bottom: the residuals of each linear regression plotted against standard length. A representative specimen from each clade is shown. A diagram depicting the phylogenetic relationships between the clades is presented in the top right. **b)** Mean absolute residuals from the linear regressions in (a), shown per clade. **c)** Mean of the absolute residuals normalized by the predicted values from the linear regression.

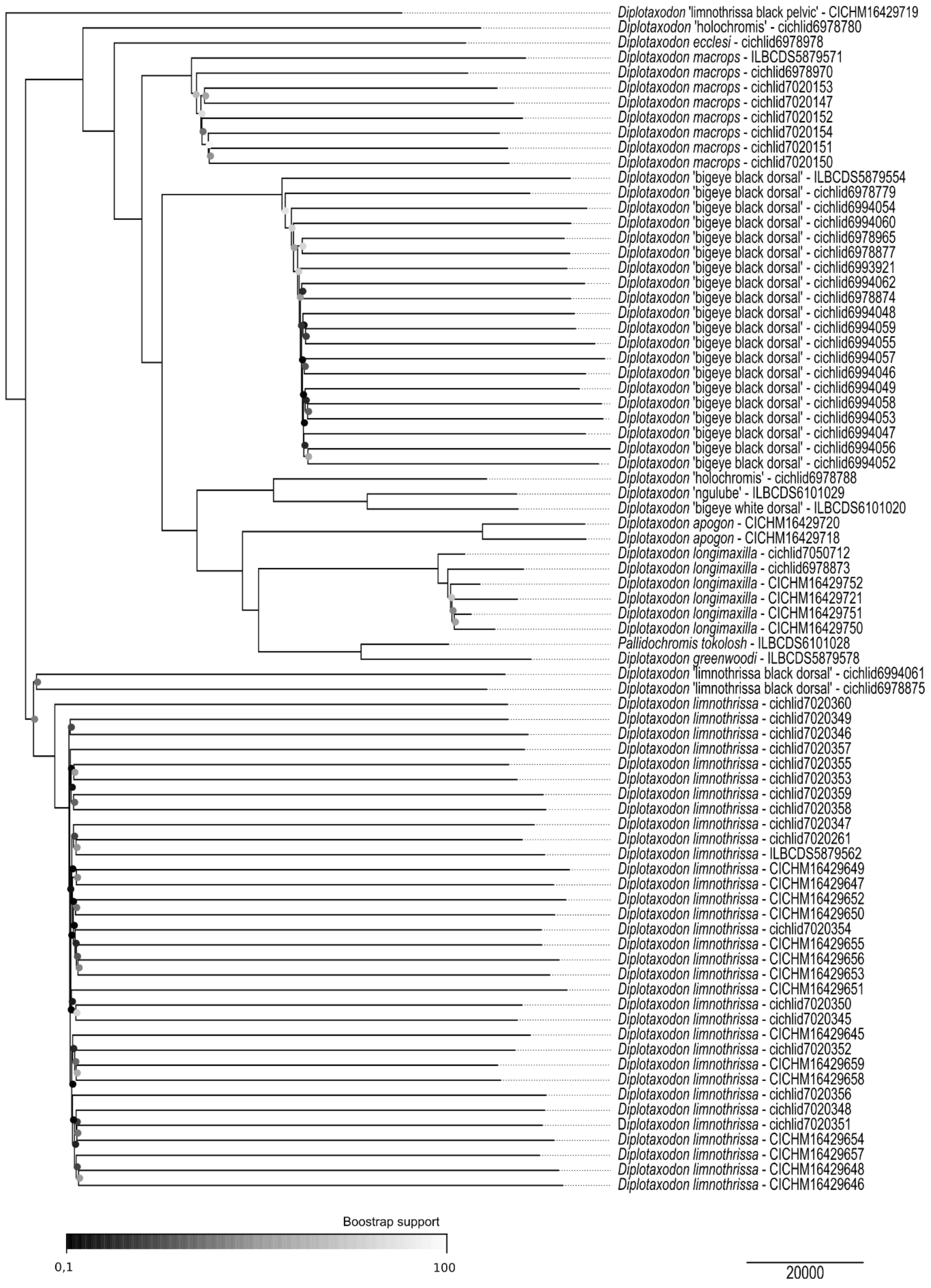

Figure S2. *Diplotaxodon* samples phylogeny. Neighbor-joining (NJ) phylogeny of 79 *Diplotaxodon* samples based on whole-genome sequencing data. The two sequenced samples morphologically identified as *D.* 'holochromis' clustered separately from each other and were therefore treated as separate species in this study (*D.* 'holochromis 1': cichlid6978780; *D.* 'holochromis 2': cichlid6978788). Bootstrap support is shown for nodes with <100% support.

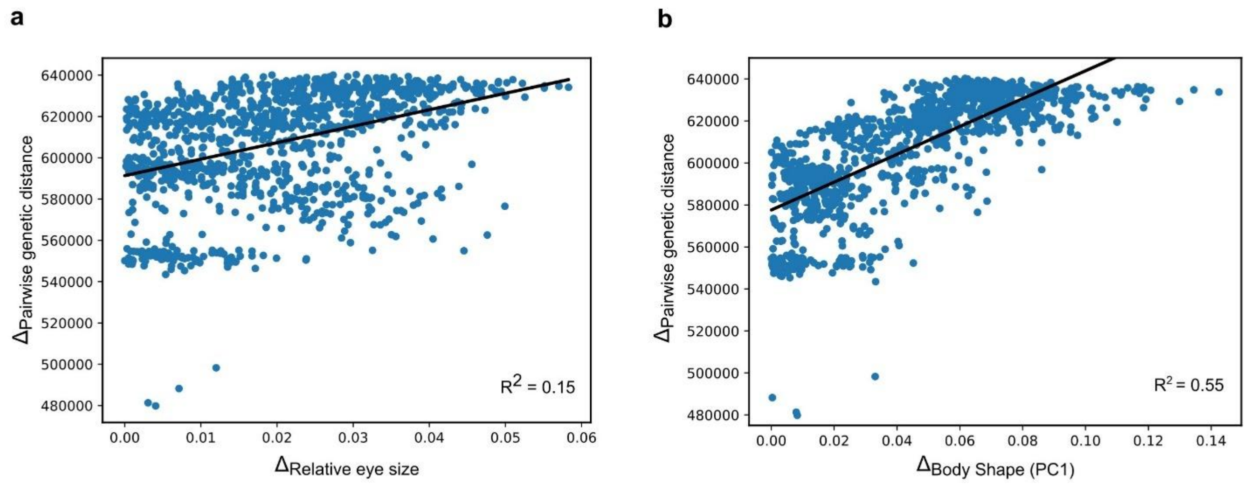

Figure S3. Eye size (a) and body shape (b) differences plotted against pairwise genetic distance in *Diplotaxodon*. The differences in genetic distance (y-axis) and in relative eye size and body shape (x-axis) were calculated for all pairwise sample comparisons among species (number of comparisons = 1,125). In (b), body shape differences are expressed as differences in absolute PC1 from a principal component analysis of the Procrustes coordinates of 18 homologous body landmarks (see Fig. S7).

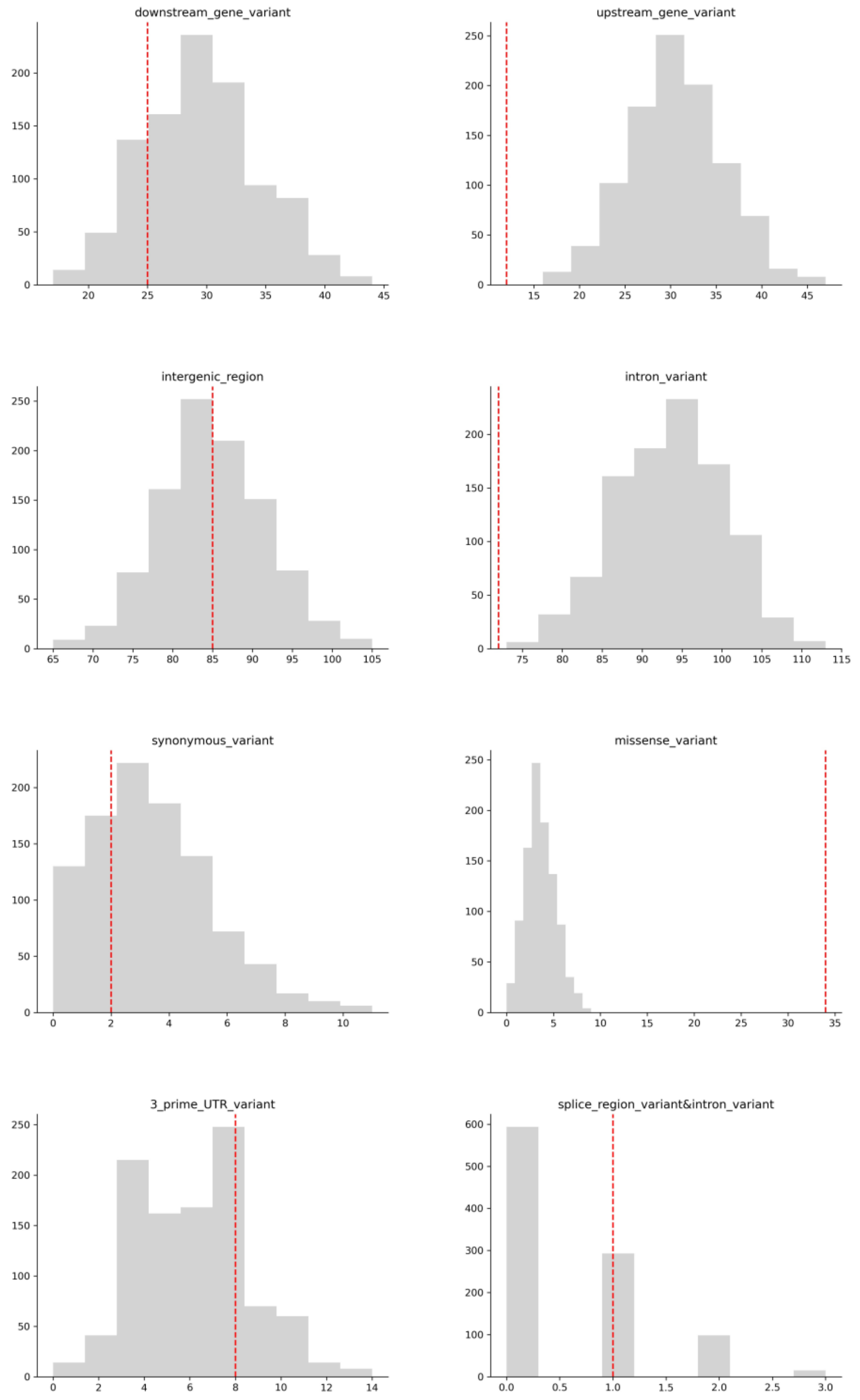

Figure S4. Empirical distributions of SNP annotations from 1,000 random draws of SNPs (grey). Annotations were counted in randomly drawn sets of 190 SNPs, with probabilities weighted by the GWA outlier allele frequency spectrum to match minor allele frequency distributions between candidate and control SNPs. The x-axis shows the number of SNPs assigned to each annotation, and the y-axis represents the number of draws. The vertical red line indicates the observed number of SNPs per annotation in the set of GWA outliers.

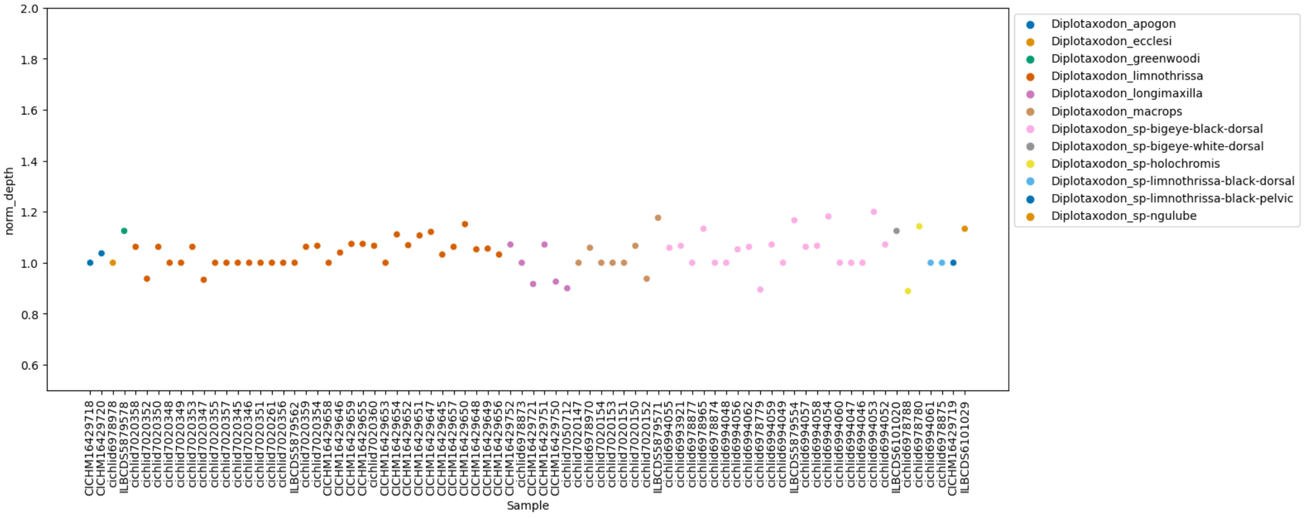

Figure S5. Median coverage per sample across the *arr3a* (LOC113031132) gene region normalized by the median genome-wide coverage.

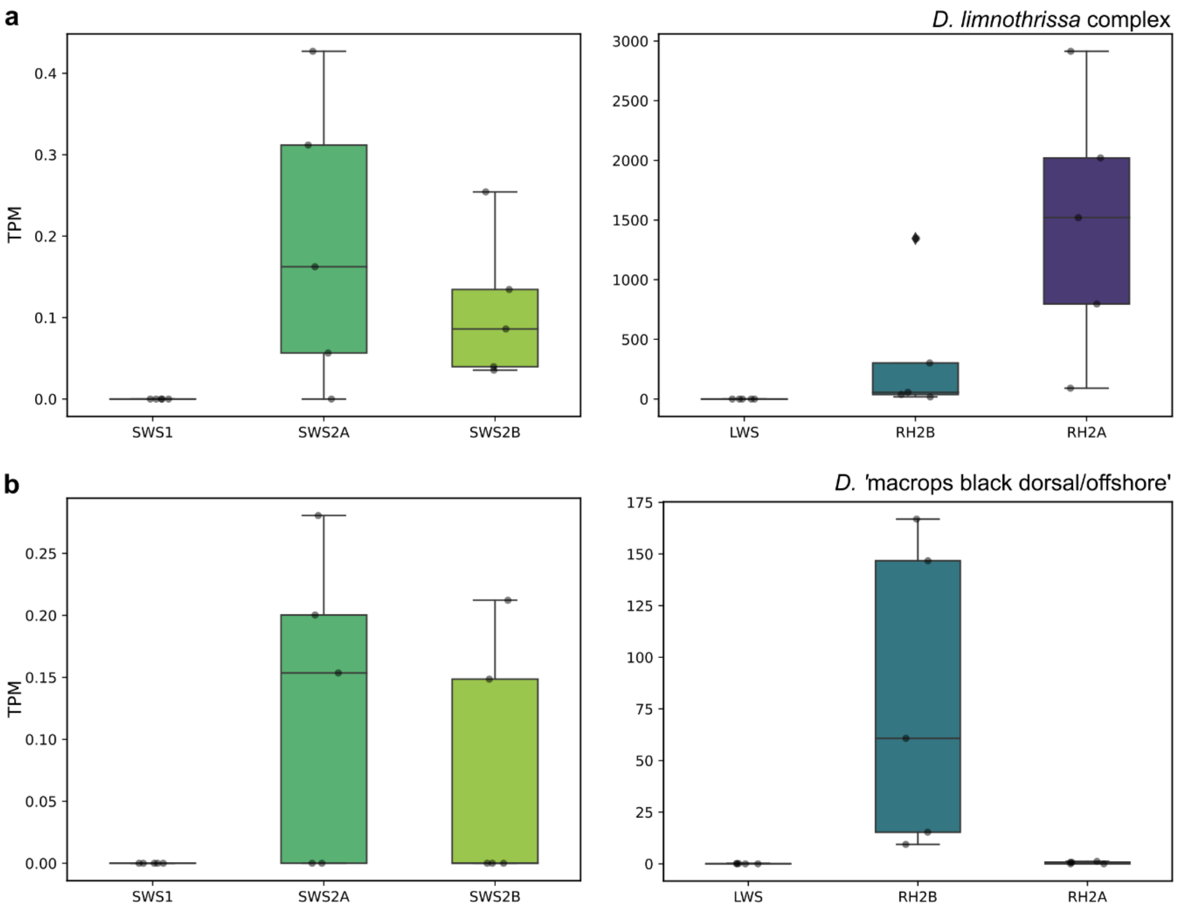

Figure S6. Normalized cone opsin expression (transcripts per million, TPM) in *D. limnothrissa* complex (**a**) and *D.* 'macrops black dorsal/offshore' (**b**) based on whole-eye transcriptomes (N = 5 per species group). Left panels show expression of single cone opsins (SWS1, SWS2a and SWS2b) and right panels show double cone opsins (RH2A, RH2B and LWS), where RH2A includes reads mapping to RH2Aα (LOC113022253) and RH2Aβ (LOC113022256). The box indicates the first and third quartiles; the central line, the median; and the whiskers, the minimum and maximum values. Outliers (values outside of 1.5x the interquartile range), are represented as diamonds.

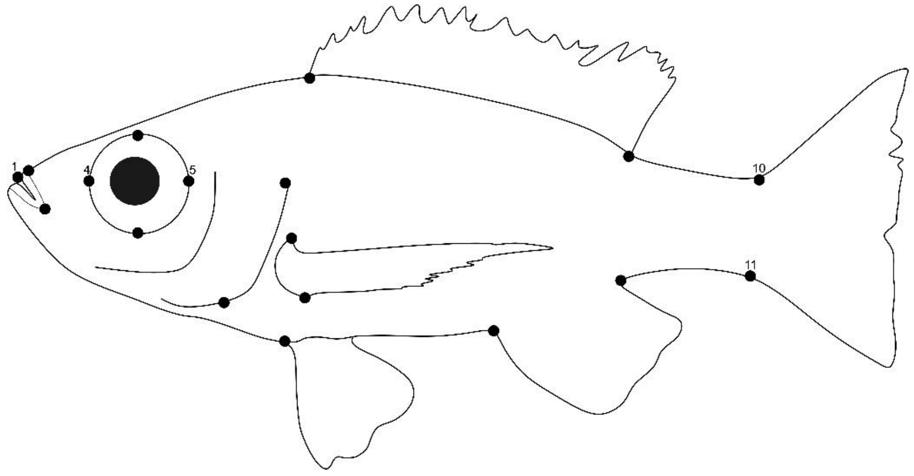

Figure S7. Homologous landmark points used in this study. The landmarks relevant for obtaining relative eye size are numbered: first, eye diameter (ED) was calculated as the distance between the coordinates for landmarks 4 and 5; standard length (SL), as the distance between the landmark 1 and the midpoint between 10 and 11. Relative eye size was then calculated as the ratio of ED to SL. For the analysis of body shape variation, coordinates of all 18 landmarks shown were used.
