# Supplementary material for: Widespread Genetic Signals of Visual System Adaptation in Deepwater Cichlid Fishes
Source: Mol Biol Evol. 2025 Jun 9;42(7):msaf147. doi: 10.1093/molbev/msaf147 (PMC12210959; doi:10.1093/molbev/msaf147)
Supplement: msaf147_Supplementary_Data [file msaf147_supplementary_data.zip › Supplementary_Data_file_description.pdf]

Description of supplementary data files

File name: Supplementary Data 1

Description:

Metadata of all samples included in this study for the analysis of eye size variation in the Lake Malawi cichlid radiation, genome-wide association and genomics analysis. Measurements of standard length (“SL”) and eye diameter (“ED”) are included for samples used in the analysis of eye size variation. Sequenced samples included in this and/or other studies have a BioSample ID associated.

File name: Supplementary Data 2

Description:

Table summarizing the number of samples per species and ecomorphological group from Supplementary Data 1 that were used in the study of eye size variation in the Lake Malawi cichlid radiation (Figure 1a, Supplementary Figure 1).

File name: Supplementary Data 3

Description:

Sampling and sequencing metadata of all *Diplotaxodon* samples used in this study, excluding RNA sequencing samples (see Supplementary Data 4). It is specified whether the samples were used in the GWAS or not (column ‘GWAS’). All samples included in this table can also be found in Supplementary Data 1.

File name: Supplementary Data 4

Description:

Metadata associated with the *Diplotaxodon* RNA sequencing samples used in the analysis of differential gene expression and the calculation of relative opsin expression in this study.

File name: Supplementary Data 5

Description:

Annotation of the top 0.01% outlier SNPs (N = 190) associated with relative eye size in 9 *Diplotaxodon* species. Includes the significance values from the likelihood ratio test, as output by GEMMA v0.98. SNPs with genome-wide significance (Bonferroni FWER < 0.05) highlighted in bold. Variants were annotated with snpEff v5.1 using the prebuilt database for the *Astatotilapia calliptera* reference genome fAstCal1.2 (GCA_900246225.3; GenBank assembly). The column “ENSEMBL GENE NAMES” lists gene symbols, when available, for genes with an ensemble ID.
